# Physiological and Skin Microbiome Divergence Among Closely Related Anurans Co‐Occurring in Agricultural Wetlands

**DOI:** 10.1002/ece3.73944

**Published:** 2026-07-10

**Authors:** Ji‐Eun Lee, Jun‐Sung Kim, Yuno Do, Jun‐Kyu Park

**Affiliations:** ^1^ Department of Biological Sciences Kongju National University Gongju Republic of Korea

**Keywords:** agricultural wetlands, congeneric comparison, conservation physiology, physiological traits, skin microbiome, trait‐based conservation

## Abstract

Understanding why endangered amphibian species decline while closely related congeners persist remains a central challenge in conservation biology. Host physiological traits and symbiotic microbial assemblages are increasingly recognized as important mediators of species responses to environmental conditions. Unlike broad comparative studies across geographically separated populations, we compared physiological capacity and skin microbiome characteristics among four anuran species, two endangered species and their respective common congeners from two genera (*Dryophytes* and *Pelophylax*), at a fine sympatric scale within shared agricultural wetlands in South Korea. Physiological traits, including body size, corticosterone levels, and bacterial killing ability, were structured primarily at the genus level, with species identity explaining 49.6% of multivariate physiological variation. Skin bacterial alpha diversity tended to be higher in common species, although statistically significant differences were not maintained after correction. Skin bacterial community composition also differed significantly among species (PERMANOVA, *R* ^2^ = 0.296), whereas *Bd* prevalence remained comparable across species (75%–85.7%). Microbial network analysis revealed species‐specific differences in topology, with highly connected networks in *D. japonicus* , fragmented structure in *D. suweonensis* , and intermediate connectivity in both *Pelophylax* species. Functional prediction analyzes suggested differences in predicted microbial functions among host species. Together, these findings suggest subtle but structured trait differentiation among sympatric species and support integrating physiology, skin microbiomes, *Bd* infection, and predicted microbial functions as a complementary trait‐based framework for amphibian conservation assessment.

## Graphical abstract

We compared physiological traits and skin microbiota of four closely related anuran species co‐occurring in agricultural wetlands, including two threatened species and two common congeners, under shared environmental conditions. We quantified body size, endocrine status, innate immune function, and *Batrachochytrium dendrobatidis* infection, together with skin bacterial diversity and community composition. By integrating host physiology and microbiome profiles, we show that shared agricultural wetland environments structure trait patterns among sympatric frogs more strongly than conservation status.

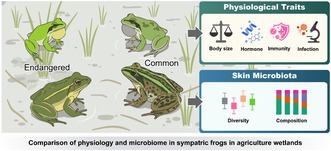

## 1 Introduction

Amphibians are currently experiencing one of the most severe global biodiversity crises, with population decline and extinctions reported across nearly all continents (Luedtke et al. 2023). Historically, the major drivers of these declines have included habitat loss and degradation, climate change, invasive species, and emerging infectious diseases (Stuart et al. 2004). More recently, additional large‐scale disturbances, such as wildfires, have been recognized as important contributors, further intensifying the pressure on amphibian populations (Beranek et al. 2023). Beyond these well‐established drivers, additional factors are likely to contribute to the decline in amphibian populations. In natural habitats, multiple stressors interact in a complex and context‐dependent manner (Blaustein and Kiesecker 2002; Rohr et al. 2008; Hof et al. 2011). Consequently, the decline in amphibian populations may be dynamic rather than static and may vary across space and time (Grant et al. 2016; Falaschi et al. 2019). Population decline may occur when the pace, magnitude, or combination of environmental changes exceeds the capacity of species‐specific physiological, behavioral, or ecological responses to track these changes (Blaustein et al. 2012; Rollins‐Smith 2017). Therefore, the relative importance of environmental stressors strongly depends on the species‐specific ecological traits and physiological tolerance ranges that modulate biological responses to changing conditions (Liu, Rowley, et al. 2021; Edwards et al. 2025). At the fundamental level, environmental tolerance is underpinned by physiological capacity (Walls and Gabor 2019). Accordingly, sustained efforts to characterize intrinsic physiological traits are essential for understanding why some amphibian populations persist while others decline, as well as for elucidating how population‐ and community‐level responses emerge within shared habitats (Walls and Gabor 2019; Park and Do 2023).

Recent studies have demonstrated that the physiological state of the host and symbiotic microbial communities interact dynamically, forming an integrated system that influences organism performance (Lynch and Hsiao 2019). Symbiotic microbiota are closely linked to host health, disease susceptibility, and immune function and can mediate host responses to environmental stressors (Hou et al. 2022; Fontaine and Kohl 2023; Lee et al. 2024; Park and Do 2024; Madison et al. 2025). Several studies have demonstrated associations between environmental factors, such as climatic conditions or pathogen exposure, and variations in host physiology and microbial richness (Catenazzi et al. 2014; Knapp et al. 2016; Kueneman et al. 2019). Because skin‐associated microbial communities and host physiological condition can influence susceptibility to infectious diseases through immune defense and barrier function, disruption of these host‐associated traits may alter vulnerability to pathogens such as *Batrachochytrium dendrobatidis* (*Bd*), making disease‐related measures useful complementary indicators of host condition (Kueneman et al. 2019).

These broad comparative approaches have provided an important context for amphibian conservation by highlighting the potential role of physiological traits and microbial diversity in shaping tolerance to environmental stress, and ultimately, population persistence (Neely et al. 2023; Woodhams et al. 2023). Building on these advances, a crucial next step is to narrow such insights to relatively finer spatial scales where ecological processes operate within local populations and assemblages (Wiens 1989; Levin 1992). At these relatively finer spatial scales, the integration of physiological and microbiological perspectives is particularly valuable for identifying subtle species‐specific vulnerabilities that may be obscured in broad‐scale comparisons. Despite the recognized potential for tight linkages between symbiotic microbial diversity and host physiological and immunological states, integrative approaches that simultaneously examine these components in wild amphibian assemblages remain limited (Rollins‐Smith 2017).

From a conservation perspective, there is growing recognition that understanding species persistence requires moving beyond coarse habitat‐ or status‐based classifications to trait‐based frameworks that explicitly incorporate functional characteristics and niche processes (Gallagher et al. 2021). Trait‐based conservation emphasizes how the physiological, morphological, and ecological attributes of species mediate their responses to environmental changes, linking fundamental and realized niches to population‐ and community‐level outcomes (Soberón and Arroyo‐Peña 2017). Integrating physiological and microbiological traits into this perspective provides a powerful means of connecting broad‐scale environmental drivers with the mechanistic processes that ultimately shape vulnerability, resilience, and coexistence in amphibian assemblages (Rollins‐Smith 2017). Within this framework, comparisons between closely related species that co‐occur within sympatric habitats are particularly informative. Species that share the same habitat are exposed to similar abiotic conditions, pathogen pools, and anthropogenic pressures, allowing intrinsic functional differences to be evaluated against a common environmental background (Richardson 2002). Under such shared conditions, divergence in physiological traits or skin microbiome structure is more likely to reflect subtle differences in life‐history strategies, microhabitat use, and host–microbe interactions rather than broad environmental filtering (Hernández‐Gómez and Hua 2023). These fine‐scale differences may represent hidden axes of vulnerability or resilience that are not apparent in large‐scale comparisons but can strongly influence how species respond to disturbances and how community‐level responses emerge.

**FIGURE 1:**
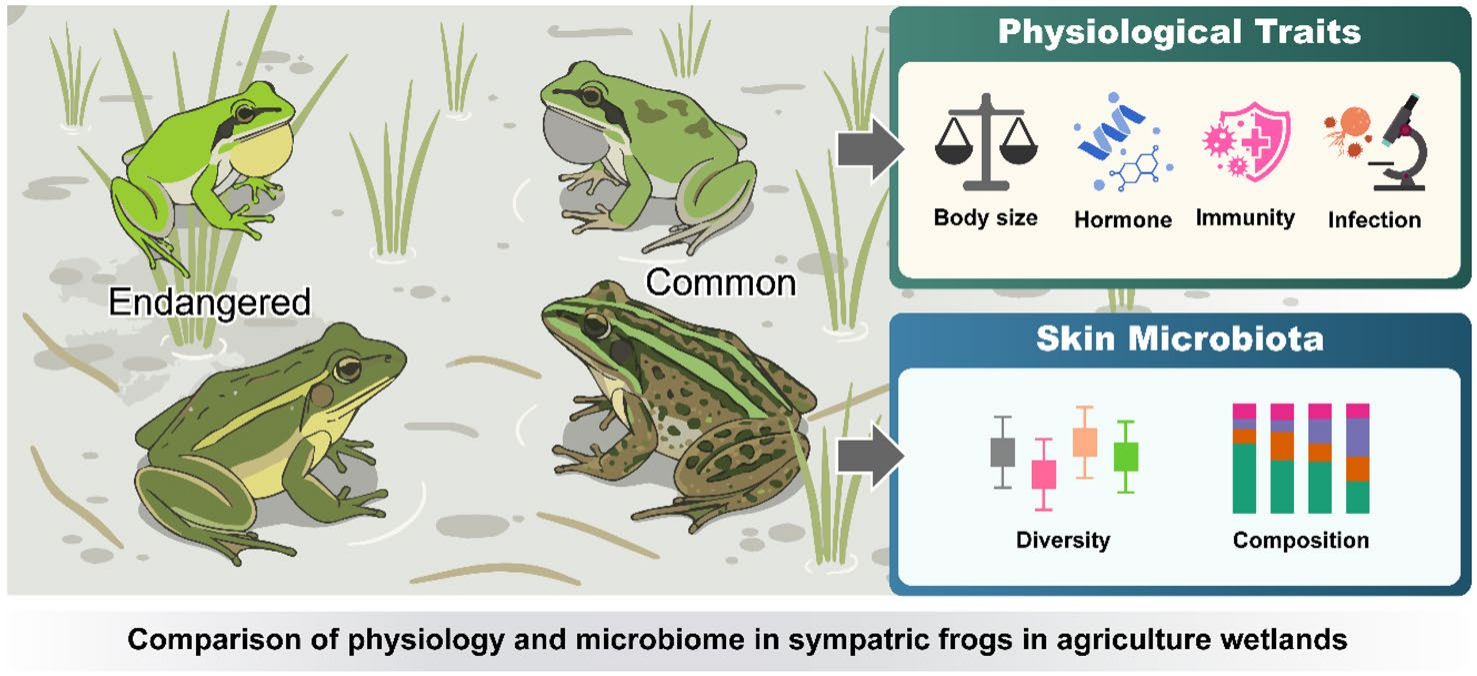
Schematic overview of the comparative framework used to examine physiological traits and skin microbiota of four closely related anuran species co‐occurring in agricultural wetlands. Two threatened species and two common congeners were compared under shared environmental conditions. Physiological traits included body size, endocrine status, innate immune function, and *Batrachochytrium dendrobatidis* (*Bd*) infection status, while skin microbiota was characterized by bacterial diversity and community composition.

Here, we adopted this fine‐scale, trait‐based approach to examine the physiological indicators, innate immune function, *Bd* infection, and skin microbiome characteristics of four closely related anuran species that co‐occur within the same agricultural wetland habitat. Rather than framing comparisons primarily around conservation status, we focused on how sympatric congeners diverge in integrated physiological and microbial profiles while occupying identical environments. Two of the focal species are currently threatened, whereas the other two are widespread, allowing conservation status to be considered an emergent property of functional similarity or divergence rather than a defining axis of comparison. By integrating univariate and multivariate analyzes, we investigated whether closely related species in sympatry exhibit subtle physiological or microbiome differentiation that may underlie differential sensitivities to environmental change, and how such patterns relate to community‐level perspectives on amphibian conservation in modified wetland landscapes (Figure 1).

We hypothesized that species with comparatively more restricted distributions would exhibit reduced physiological capacity, lower microbial diversity, and less stable microbial community structure compared with their widespread sympatric congeners despite occupying shared environmental conditions. Specifically, we predicted that (1) these species would show lower physiological performance and reduced skin microbial diversity, (2) microbial community structure would exhibit lower connectivity and reduced stability relative to widespread congeners, and (3) disease‐related indicators would reflect interspecific variation in host physiological and microbial condition.

## 2 Materials and Methods

### 2.1 Study Species and Field Sampling

We focused on two endangered anuran species in Korea, the Suweon tree frog ( *Dryophytes suweonensis* ) and gold‐spotted pond frog ( *Pelophylax chosenicus* ), and compared them with two closely related, widespread congeners, the common tree frog ( *Dryophytes japonicus* ) and black‐spotted pond frog ( *Pelophylax nigromaculatus* ). The two common species are widely distributed across the Korean Peninsula (Lee et al. 2025), whereas *Dryophytes suweonensis* and *Pelophylax chosenicus* show comparatively more restricted distributions (Borzée et al. 2018). Those four species share a similar breeding phenology and frequently co‐occur in rice paddies. We selected two rice paddy complexes where all four species were present and conducted field sampling. To protect the endangered populations, precise geographic coordinates have not yet been reported.

Fieldwork was conducted in June 2024 during the peak breeding season when males of all four species were actively calling. The investigations were conducted between 21:00 and 23:00, which corresponds to the main activity period of the target species. No other amphibian species were observed in the paddy fields surveyed during the sampling period.

Fieldwork was conducted by two teams operating in parallel. Within each team, one person was responsible for capturing frogs using sterile nitrile gloves, while the other performed physiological and microbiological sampling to minimize handling time. Individuals were captured manually using sterilized nitrile gloves. For each species, we initially collected 12 adults (7 males and 5 females). Immediately after capture, the individuals were sampled for salivary corticosterone (CORT), innate immune function, body size, *Bd* infection load, and skin bacterial communities. Individuals with insufficient blood volume, hemolysis, or contaminated saliva samples were excluded from subsequent analyzes. Consequently, the final sample sizes used in the analyzes were eight individuals (five males and three females) of the common treefrogs, 9 (5 males and 4 females) of the Suweon treefrogs, 7 (3 males and 4 females) of the black‐spotted pond frogs, and 8 (5 males and 3 females) of the gold‐spotted pond frogs.

All procedures involving endangered species were conducted with the appropriate collection and sampling permits (ED202403ECP0009). All animal experimental procedures and laboratory animal management were approved by the Laboratory Animal Experiment Ethics Committee of Kongju National University (KNU_2024‐01) and conducted in accordance with the institutional and national guidelines for the care and use of animals in research.

### 2.2 Physiological and Microbiological Sampling

Field sampling was conducted to minimize handling time and stress (Romero and Reed 2005; Titon et al. 2020). Immediately after capture, saliva was collected in the field within 3 min using pre‐weighed cotton pads placed in the buccal cavity to minimize handling‐induced elevation of CORT levels. Salivary cotton pads (PURENAIL, Gillingham, Kent, UK) were cut in the laboratory to a size that fit into 1.5 mL microcentrifuge tubes and weighed to the nearest 0.001 g using a digital scale; these baseline cotton weights were recorded. In the field, the sampler gently opened the frog's mouth with a sterile pipette tip and placed the pre‐weighed cotton pad inside the buccal cavity for 1 min. Saliva collection was completed within 1 min of capture to minimize stress‐related elevation of corticosterone. After sampling, the cotton pad was placed into a labeled 1.5 mL microcentrifuge tube and stored in a portable cooler at approximately −20°C.

Ventral skin was swabbed for *Bd* detection and skin microbiome analysis. Sterile Isohelix DNA swabs (SK‐2S, Isohelix, Harrietsham, UK) were used to swab the abdomen eight times, dorsal surface eight times, and each limb including the toes twice. Swab heads were broken off into sterile 2 mL microcentrifuge tubes and stored in the same cooler at −20°C. Sterile nitrile gloves were changed between individuals to avoid cross‐contamination. Frogs were gently rinsed with sterile distilled water prior to swabbing to remove loosely attached soil particles, debris, and transient environmental microorganisms from the skin surface. After field sampling, live frogs were transported to the Animal Laboratory at Kongju National University within 2 h, where the samples were frozen until processing.

To avoid anesthesia‐related mortality in endangered species, blood samples were collected via maxillary vein puncture using heparinized microcapillary tubes (Forzán et al. 2012). We did not use MS‐222 or other anesthetic agents because of the potential risk of mortality in endangered species. Approximately 25 μL of blood was collected from each individual, which is below commonly accepted safe limits for small amphibians. After blood sampling, frogs were placed individually in containers with clean water and monitored for approximately 2 h to confirm normal behavior and recovery. Blood samples were allowed to react with heparin for 1 h and were then centrifuged at 1800 *× g* for 10 min to separate plasma. The supernatant was transferred to new tubes and stored at −30°C until bacterial killing ability (BKA) analysis. The plasma was separated by centrifugation and stored for subsequent immune assays.

Snout–vent length (SVL) and body mass were measured after blood sampling. SVL was measured to the nearest 0.1 mm using a digital caliper, and body mass was measured to the nearest 0.01 g using a portable electronic balance. From capture in the field to completion of blood sampling and morphometric measurements in the laboratory, the total handling time per individual did not exceed approximately 4 h. All frogs were monitored every 2 h until the following afternoon, and individuals showing no signs of distress were released at their original capture locations. Individuals were monitored after sampling and released at their original capture locations once normal behavior was confirmed.

### 2.3 Salivary CORT Assay

Salivary CORT was quantified as an index of physiological stress. Saliva samples were collected immediately after capture to minimize the handling‐induced elevation of hormone levels (Romero and Reed 2005). Cotton pads saturated with saliva were frozen before CORT extraction to allow for the precipitation of viscous proteins. Cotton pads saturated with saliva were stored at −20°C for up to 2 weeks prior to extraction. For each sample, the cotton pad was weighed again to the nearest 0.001 g, and saliva mass was calculated by subtracting the initial cotton weight from the post‐sampling weight.

For each sample, the saliva mass was calculated by subtracting the initial cotton weight from the post sampling weight. Salivary CORT was extracted using assay buffer and clarified by centrifugation with trichloroacetic acid to reduce protein interference (Hammond et al. 2018; Park and Do 2022). Each cotton pad was placed into a perforated 1.5 mL microcentrifuge tube, and 150 μL of assay buffer supplied with the ELISA kit was added. Samples were centrifuged at 5000 × *g* for 10 min, and the supernatant was collected. Trichloroacetic acid was added at a ratio of 1.5 μL per 10 mg of saliva and centrifuged again at 3000 × *g* for 8 min to reduce interference from salivary proteins. After a 10 min reaction period, the clarified supernatant was transferred to new tubes and stored at −30°C until hormone quantification.

CORT concentrations were measured using a commercial enzyme‐linked immunosorbent assay (ELISA) kit (Cayam, Michigan, USA) following the manufacturer's instructions. Salivary corticosterone concentrations were measured using a commercial enzyme‐linked immunosorbent assay kit (Corticosterone EIA, Cayman Chemical, Ann Arbor, MI, USA). All samples were assayed in triplicate wells. Absorbance was read at 412 nm using a microplate spectrophotometer, and hormone concentrations were calculated from the standard curve. All samples were assayed in triplicate, and the final concentrations were adjusted for sample dilution and saliva mass and expressed as picograms of CORT per milligram of saliva.

### 2.4 Innate Immune Assay

Innate immune function was evaluated by measuring the bacterial killing ability (BKA) of plasma against nonpathogenic *Escherichia coli* , which reflects the complement‐mediated bactericidal capacity (Assis et al. 2013). Considering its broad use as a physiological indicator in amphibian studies, BKA was assessed using protocols established in previous anuran studies (Assis et al. 2019; Lee et al. 2025). Plasma samples were diluted in amphibian Ringer's solution and incubated with standardized bacterial suspensions.

Plasma samples (10 μL) were diluted in 190 μL of amphibian Ringer's solution (ARS). A suspension of non‐pathogenic *Escherichia coli* (Microbio‐Logics #24311, ATCC 8739, St. Cloud, MN, USA) was prepared at approximately 10^6^ cells, and 10 μL of the bacterial suspension was added to each plasma dilution. Positive controls consisted of bacterial suspension added to ARS without plasma, whereas negative controls contained ARS only. Plasma–bacteria mixtures and controls were incubated at 37°C for 1 h. After incubation, 500 μL of tryptic soy broth was added to each tube. Subsequently, 300 μL of each bacterial suspension was transferred in duplicate to 96‐well microplates and incubated at 37°C.

Following the incubations, bacterial growth was quantified spectrophotometrically, and optical density was monitored over time to capture the dynamics of bacterial growth. Optical density of positive controls was measured at one‐hour intervals to characterize growth dynamics, and values obtained during the exponential growth phase were used for BKA calculation. Bacterial growth was monitored by measuring optical density at 600 nm using a microplate spectrophotometer. Optical density readings were recorded hourly over a total period of 6 h. The BKA was calculated as the proportional reduction in bacterial growth relative to positive controls, yielding a dimensionless index of innate immune function. Bacterial killing ability was calculated as: BKA = 1 – (Optical density of the sample/Optical density of the positive control). This value represents the proportion of bacteria killed relative to the positive control and was used as an index of innate immune function.

### 2.5 Analysis of Skin Bacterial Community

Microbial DNA was extracted from skin swab samples and used for both skin microbiome analysis and quantification of *Bd* infection. DNA extraction was performed using the PrepMan Ultra Sample Preparation Reagent Kit (Applied Biosystems, Foster City, CA, USA) following the manufacturer's instructions. Samples were homogenized using a TissueLyser II (Qiagen, Hilden, Germany). DNA concentration and integrity were assessed using a QFX fluorometer (DeNovix Inc., Wilmington, DE, USA) and agarose gel electrophoresis. The negative controls consisted of sterile cotton swabs and extraction blanks containing nuclease‐free water. No detectable PCR amplification was observed in any of the negative controls, indicating a negligible risk of contamination. Consequently, no further analyzes of the negative control samples were performed.

The V4 hypervariable region of the bacterial 16S rRNA gene was amplified using primers with Illumina overhang adapter sequences: 515F (5′‐TCGTCGGCAGCGTCAGATGTGTATAAGAGACAGGTGCCAGCMGCCGCGGTAA‐3′) and 806R (5′‐GTCTCGTGGGCTCGGAGATGTGTATAAGAGACAGGGACTACHVGGGTWTCTAAT‐3′). PCR amplification was conducted using KAPA HiFi HotStart ReadyMix (Kapa Biosystems Inc., Wilmington, MA, USA), following the Illumina protocol “Preparing 16S Ribosomal RNA Gene Amplicons for the Illumina MiSeq System” with minor adjustments (Illumina 2011). Primary PCR products were purified using the Agencourt AMPure XP system (Beckman Coulter). A second PCR was performed to add dual indices and sequencing adapters using the Nextera XT Index Kit (Illumina). Indexed amplicons were purified again using AMPure XP, and the concentration and quality of the final libraries were assessed by fluorometry and electrophoresis. Equimolar amounts of each library were pooled, diluted to the appropriate loading concentration, and sequenced on an Illumina MiSeq platform (Illumina, San Diego, CA, USA) in paired‐end mode.

Raw paired‐end FASTQ reads were processed using QIIME2 version 2022.2 (Bolyen et al. 2019). Amplicon sequence variants (ASVs) were inferred using the DADA2 pipeline, which includes quality filtering, denoising, and chimera removal (Callahan et al. 2016). Trimming and truncation parameters were selected based on per‐base quality profiles visualized using the Demux plugin. Taxonomic assignment was performed using a classifier trained with the SILVA reference database (Pruesse et al. 2007). Only bacterial sequences were retained for downstream analyzes. Operational taxonomic units (OTUs) were defined at 97% sequence similarity where required for specific community‐level analyzes. skin bacterial community structure was characterized using relative abundance profiles, α‐diversity indices, and β‐diversity metrics, which were calculated in R using the microeco package (version 3.4.4) (Liu, Cui, et al. 2021).

### 2.6 Microbial Network and Functional Analyzes

To further characterize skin bacterial communities beyond taxonomic composition and diversity, we performed species‐specific microbial co‐occurrence network analysis and predicted functional profiling. For network analysis, bacterial abundance tables generated from the QIIME2‐processed ASV data were agglomerated at the genus level. Low‐abundance and low‐prevalence genera were removed before network construction to reduce unstable associations driven by rare taxa. Networks were constructed separately for each host species using genus‐level relative abundance profiles. Pairwise associations among bacterial genera were estimated using rank‐based correlations, and only stable associations that passed the predefined correlation and bootstrap support filters were retained. Positive and negative edges were interpreted as co‐occurrence and co‐exclusion patterns, respectively, rather than direct microbial interactions. For each species‐specific network, nodes represented bacterial genera and edges represented retained associations. Network‐level properties, including number of nodes, number of edges, network density, mean degree, clustering coefficient, modularity, and number of connected components, were calculated to compare microbiome association structures among host species.

Predicted microbial functional potential was inferred using PICRUSt2 from the ASV representative sequences and the corresponding feature table (Douglas et al. 2020). The QIIME2 feature table and representative sequences were exported as BIOM and FASTA files and used as PICRUSt2 inputs. ASVs that could not be sufficiently aligned or placed against the PICRUSt2 reference phylogeny were excluded according to the placement quality criteria. Predicted enzyme commission profiles were first inferred and then used to estimate MetaCyc pathway abundance profiles (Caspi et al. 2020). We analyzed unstratified EC and MetaCyc pathway abundance profiles because the objective was to compare community‐level functional potential among host species rather than to attribute individual pathways to specific ASVs.

For downstream analysis, predicted EC and MetaCyc pathway abundance tables were converted to relative abundance profiles. Bray–Curtis dissimilarities were calculated among samples, and principal coordinates analysis was used to visualize differences in predicted functional composition among host species. Species effects on predicted functional profiles were evaluated using PERMANOVA. To identify predicted pathways contributing most strongly to species‐level differences, each MetaCyc pathway was compared among the four host species using Kruskal–Wallis tests, followed by false‐discovery‐rate correction. The top species‐associated predicted pathways were visualized using species‐level mean relative abundance and log2 enrichment relative to the global mean. Original MetaCyc pathway identifiers were retained in the Supporting Information Table, whereas pathway names were used in the main figure for readability.

Because the functional profiles were inferred from 16S rRNA gene amplicon data rather than shotgun metagenomic or metatranscriptomic data, all functional results were interpreted as predicted functional potential. Similarly, microbial networks were interpreted as descriptive association structures and not as evidence of direct ecological interactions among bacterial taxa (Röttjers and Faust 2018).

### 2.7 Detection and Quantification of *Bd* Infection

To assess *Bd* infection load, we performed real‐time quantitative PCR (qPCR) on DNA extracted from skin swabs. TaqMan‐based assays were performed in triplicate for each sample. We targeted the ITS1–5.8S–ITS2 region of *Bd* using a primer–probe set based on published protocols: forward primer ITS1‐3 Chytr (5′‐CCTTGATATAATACAGTGTGCCATATGTC‐3′), reverse primer Chytr 5.8S, and a TaqMan probe (Chytr MGB2:5′‐FAM‐CGAGTCGAACAAAAT‐MGBNFQ‐3′), following previous studies with minor modifications (Boyle et al. 2004; Hyatt et al. 2007).

Each reaction was performed in a total volume of 20 μL, containing 10 μL of TaqMan Universal PCR Master Mix 2X (Applied Biosystems, Waltham, USA), 900 nM of each primer, 250 nM probe, and 5 μL of template DNA (diluted 1:10 in nuclease‐free water). Amplification was performed on a Bio‐Rad CFX Duet Real‐Time PCR System (Bio‐Rad Laboratories, Hercules, CA, USA) under the following conditions. The qPCR program consisted of an initial denaturation at 95°C for 10 min, followed by 50 cycles of 95°C for 15 s and 60°C for 1 min, with fluorescence measured at the end of each extension step. Reactions were held at 4°C following amplification. For absolute quantification, we included a previously validated positive control derived from *Bd*‐infected fire‐bellied toad ( *Bombina orientalis* ) tissue, along with a negative cont. rol of nuclease‐free distilled water (Park et al. 2025). Quantification cycle (C_q_) values were calculated using the Bio‐Rad CFX Maestro Software (version 2.3). Following WOAH recommendations, samples were considered *Bd*‐positive when a characteristic amplification curve was detected with C_q_ ≤ 39 in all three technical replicates, and *Bd*‐negative when no characteristic amplification was observed with C_q_ > 41 in all three replicates (Hyatt 2017). Samples with C_q_ values between 39 and 41 or inconsistent replicate amplifications were treated as indeterminate and retested. *Bd* load was subsequently estimated from Cq values using a previously described approach (Lee et al. 2025), in which differences in Cq values relative to the positive control were used to estimate relative DNA quantity assuming 100% amplification efficiency.

### 2.8 Statistical Analyzes

All statistical analyzes were performed using R version 4.5.2 (Team 2016). Differences in physiological variables (SVL, body mass, salivary CORT levels, and BKA levels) and microbiome α‐diversity indices among species were tested using Kruskal–Wallis tests, followed by Dunn's post hoc tests with Holm–Bonferroni adjustment when appropriate.

Differences in skin bacterial community composition were evaluated using Bray–Curtis dissimilarity ordination methods, including principal coordinates analysis (PCoA) and nonmetric multidimensional scaling (NMDS), and (permutational multivariate analysis of variance) PERMANOVA, performed in R using the vegan package (Oksanen et al. 2013). Skin bacterial community composition was also visualized using stacked bar plots of relative abundance.

To examine multivariate physiological patterns, we conducted principal component analysis (PCA) using body size, endocrine, immune, and *Bd* infection variables and evaluated species differences and dispersion using PERMANOVA. PCA was conducted using five variables: body weight, snout–vent length, bacterial killing ability, salivary corticosterone, and *Bd* infection (Cq values). Species differences and within‐species dispersion in multivariate physiological space were additionally evaluated using tests of multivariate dispersion.

Finally, redundancy analysis (RDA) was performed to integrate physiological variables, microbiome diversity indices, and representative bacterial taxa, and the joint gradients linking host physiology, microbiome structure, and species identity were explored. The RDA included five physiological variables, two microbiome diversity indices (Shannon index and phylogenetic diversity), and the relative abundances of six representative bacterial genera (*Verticiella*, *Rhizobacter*, *Enhydrobacter*, *Comamonas*, *Deinococcus*, and *Luteolibacter*).

## 3 Results

### 3.1 Physiological Differences Among the Four Species

**FIGURE 2:**
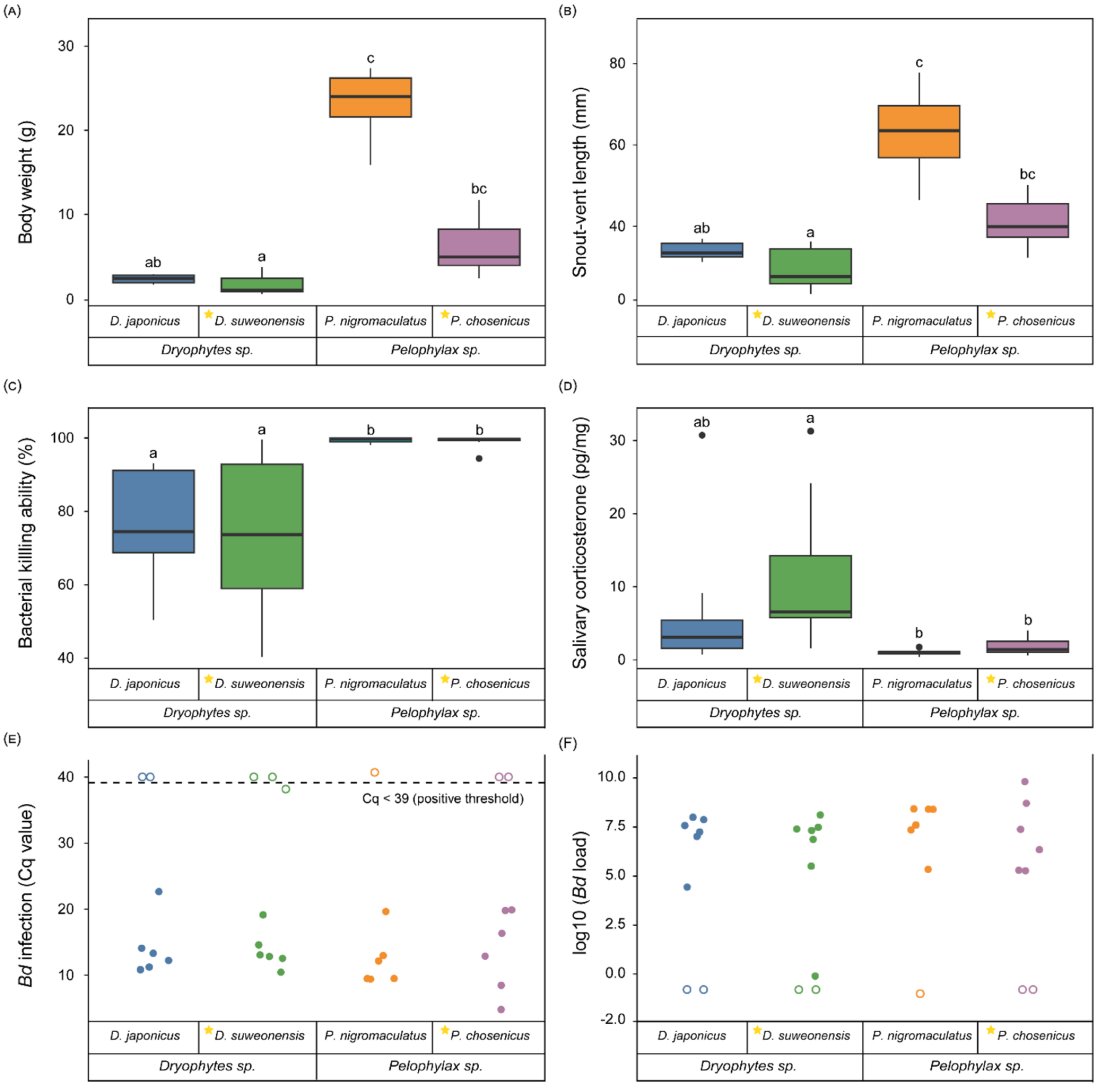
Differences in morphology, physiology, and *Bd* infection among four frog species: (A) body weight, (B) snout–vent length (SVL), (C) bacterial killing ability (BKA), (D) salivary corticosterone (CORT), (E) *Bd* infection intensity (Cq values), and (F) *Bd* infection load (log_10_‐transformed *Bd* load). Different letters indicate significant differences among species (*p* < 0.05). The dashed line in panel (E) denotes the *Bd*‐positive threshold (Cq < 39). In panel (F), filled circles indicate *Bd*‐positive individuals, and open circles indicate *Bd*‐negative individuals.

Kruskal–Wallis tests revealed significant differences among the four species in all physiological variables, including weight (*χ* ^2^ = 24.73, *p* < 0.001), SVL (*χ* ^2^ = 23.77, *p* < 0.001), BKA (*χ* ^*2*^ = 19.82, *p* < 0.001), and salivary CORT (*χ* ^2^ = 17.39, *p* < 0.001) (Figure 2).

Body mass and SVL exhibited nearly identical patterns (Figure 2A,B). *Pelophylax nigromaculatus* was significantly larger than *Dryophytes suweonensis* and *D. japonicus* (adjusted *p* < 0.05), whereas no significant differences were detected between *P. nigromaculatus* and *P. chosenicus* (adjusted *p* > 0.05). The two *Dryophytes* species did not differ significantly in body mass or SVL (adjusted *p* > 0.05).

The BKA exhibited a clear genus‐level split, with both *Pelophylax* species ( *P. nigromaculatus* and *P. chosenicus* ) showing significantly higher values than those of both *Dryophytes* species ( *D. japonicus* and *D. suweonensis* ) (adjusted *p* < 0.05) (Figure 2C). No significant differences in BKA were detected within the genera (adjusted *p* > 0.05). CORT levels were the highest in *D. suweonensis* , which were significantly higher than those in *P. nigromaculatus* and *P. chosenicus* (adjusted *p* < 0.05); however, the differences between *D. suweonensis* and *D. japonicus* , as well as between the two *Pelophylax* species, were not significant (adjusted *p* > 0.05) (Figure 2D).

*Bd* prevalence was comparable across species, with *D. japonicus* , *D. suweonensis* , and *P. chosenicus* each showing a prevalence of 75%, and *P. nigromaculatus* showing a slightly higher prevalence of 85.7% (Figure 2E).

### 3.2 PCA and Dispersion

**FIGURE 3:**
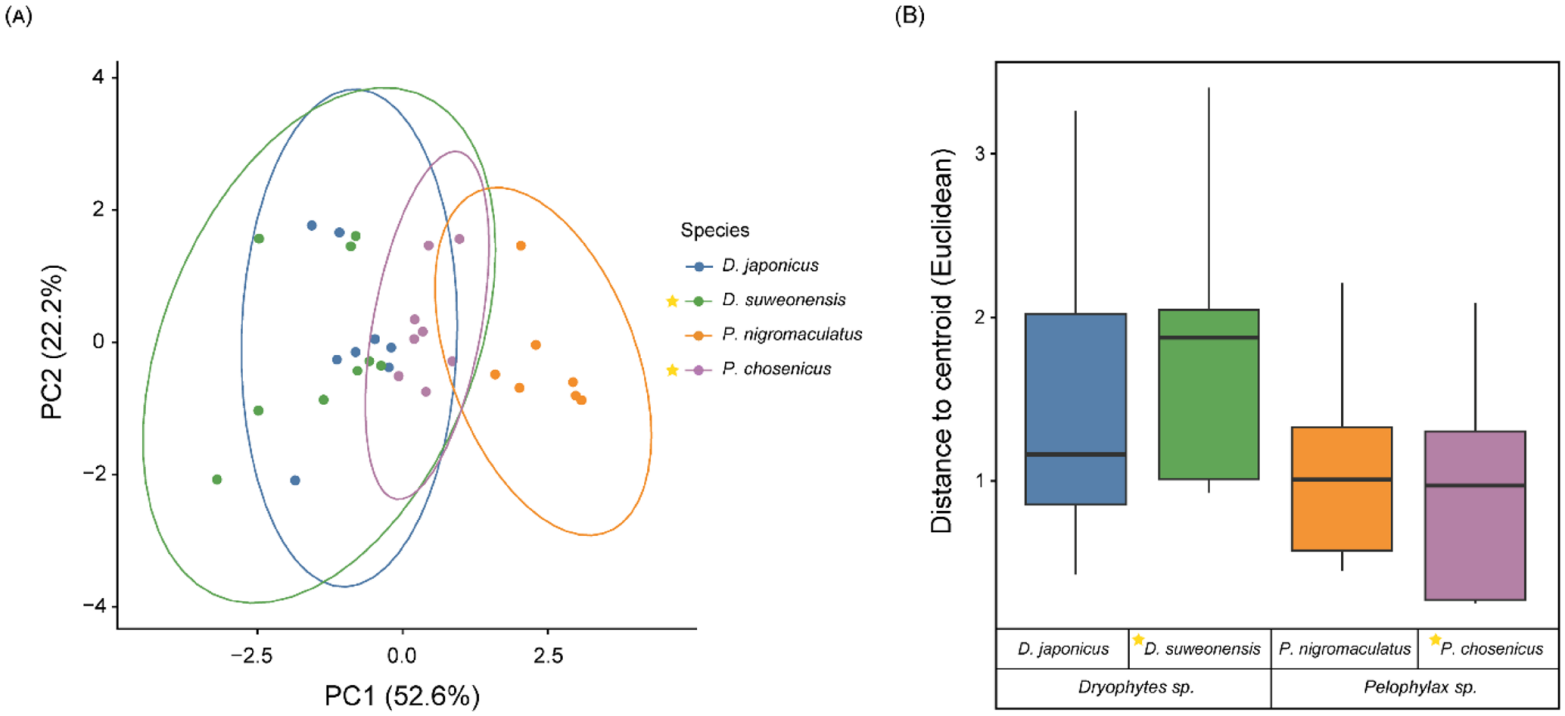
Differences in multivariate physiological patterns among four frog species: (A) PCA of physiological variables and *Bd* infection, and (B) within‐species dispersion measured as distance to centroids. Species differences were evaluated using PERMANOVA (*p* < 0.05).

In the PCA results, the first two principal components (PC1 and PC2) explained 52.6% (PC1) and 22.2% (PC2) of the total variation (Figure 3A). Along these axes, *D. japonicus* and *D. suweonensis* clustered closely, reflecting their similar physiological profiles, whereas *P. chosenicus* largely overlapped with this cluster. In contrast, *P. nigromaculatus* was distinct from the other three species and showed the greatest physiological divergence from *D. suweonensis* . PERMANOVA supported these differences, indicating that species identity significantly explained the variation in physiological traits (*F* = 9.177, *R* ^2^ = 0.4958, *p* < 0.001).

However, multivariate dispersion (distance to species centroids) did not differ significantly among species (*F* = 2.03, *p* = 0.141), suggesting that physiological variation was comparable across species (Figure 3B).

### 3.3 Microbiome Diversity

**FIGURE 4:**
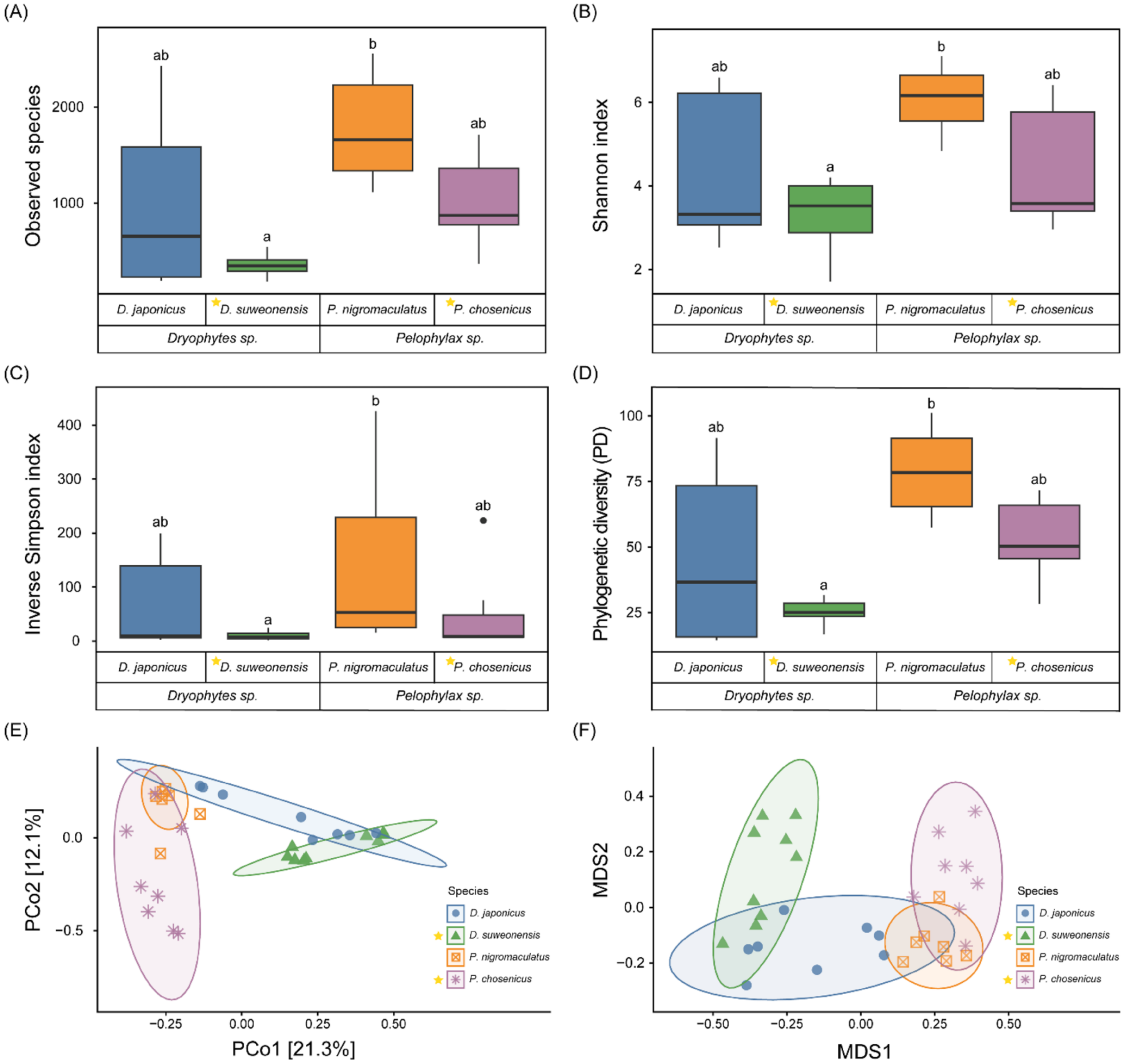
Alpha and beta diversity of skin bacterial communities across four frog species: (A) observed species richness, (B) Shannon index, (C) inverse Simpson index, (D) phylogenetic diversity, and beta diversity visualized by (E) principal coordinates analysis (PCoA) and (F) non‐metric multidimensional scaling (NMDS).

Analysis of skin microbiome α‐diversity revealed significant differences among the four species across all indices, including observed species (*χ* ^2^ = 15.278, *p* = 0.002), Shannon index (*χ* ^2^ = 10.127, *p* = 0.018), Inverse Simpson index (*χ* ^2^ = 9.187, *p* = 0.027), and Phylogenetic diversity (*χ* ^2^ = 14.548, *p* = 0.002) (Figure 4A–D).

Despite these overall differences, the pairwise patterns were highly consistent across indices. A significant difference was detected between *D. suweonensis* and *P. nigromaculatus* (adjusted *p* < 0.05). Although the two common species ( *D. japonicus* and *P. nigromaculatus* ) tended to exhibit higher α‐diversity than the two threatened species ( *D. suweonensis* and *P. chosenicus* ), no significant within‐genus differences were detected between *D. japonicus* and *D. suweonensis* or between *P. nigromaculatus* and *P. chosenicus* (all adjusted *p* > 0.05).

The β‐diversity patterns were similar to those of α‐diversity (Figure 4E,F). In the PCoA results, PCo1 and PCo2 explained 21.3% and 12.1% of the variation, respectively; however, the NMDS ordination yielded an acceptable stress value of 0.12, indicating a reliable representation of the multivariate dissimilarity structure. PERMANOVA further confirmed the significant differences in community composition among species (*F* = 3.931, *R* ^2^ = 0.296, *p* < 0.001).

Across both ordination methods, *P. nigromaculatus* and *P. chosenicus* exhibited substantial overlap, whereas *D. suweonensis* formed a distinct cluster separate from the two *Pelophylax* species. *D. japonicus* showed an intermediate pattern, partially overlapping all three species, reflecting its mixed community composition.

### 3.4 Microbiome Composition

**FIGURE 5:**
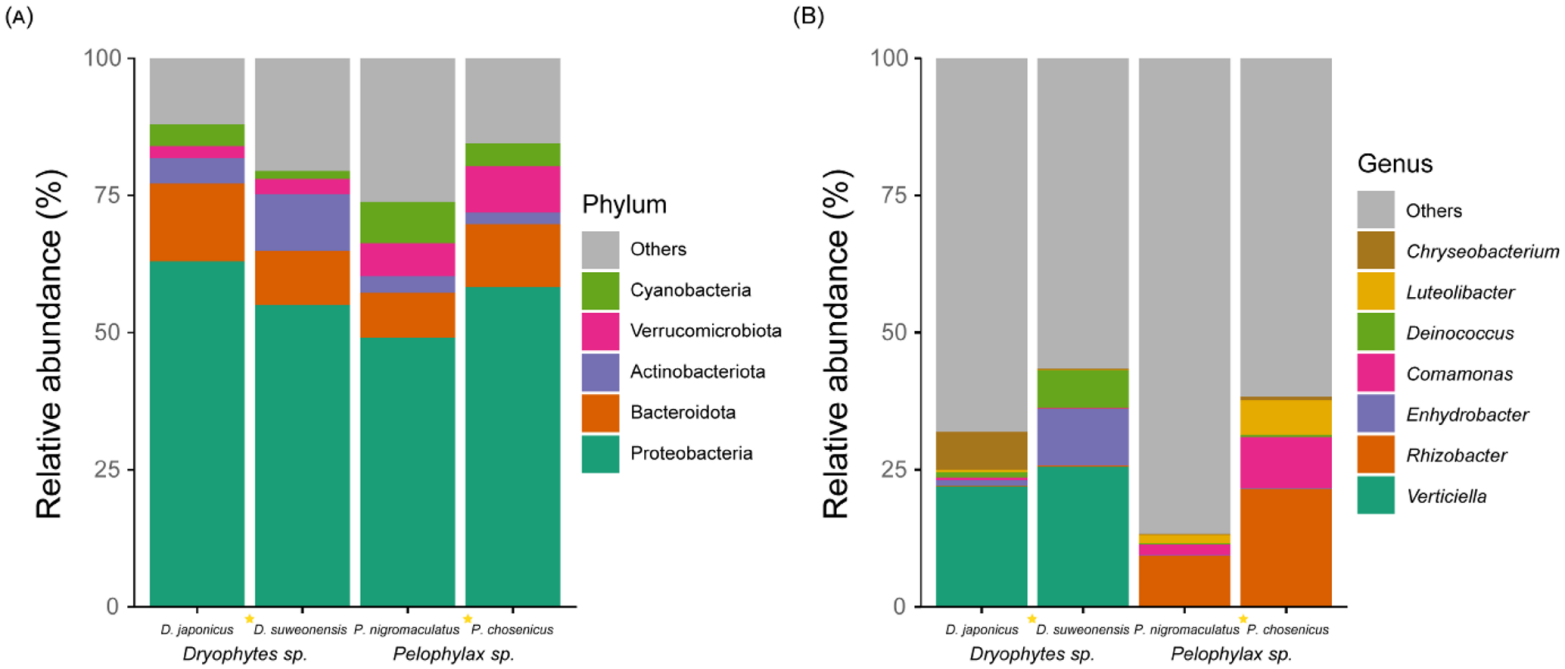
Relative abundance of skin bacterial communities across four frog species at the (A) phylum level and (B) genus level.

At the phylum level, skin bacterial communities of all four species were dominated by Proteobacteria, which accounted on average for 56.5% of total relative abundance (*χ* ^*2*^ = 2.542, *p* = 0.468), followed by Bacteroidota at 11.0% (*χ* ^2^ = 2.838, *p* = 0.417), with no significant differences among species (Figure 5A). In contrast, Actinobacteriota differed significantly among species (*χ* ^*2*^ = 15.541, *p* = 0.002). *Dryophytes suweonensis* had the highest mean relative abundance (10.4%), which was significantly higher than that of *P. chosenicus* (2.1%; adjusted *p* < 0.05), whereas *P. nigromaculatus* (3.1%) and *D. japonicus* (4.6%) did not differ significantly from the other species (adjusted *p* > 0.05).

Cyanobacteria also varied among species (*χ* ^2^ = 9.086, *p* = 0.028), with *D. suweonensis* showing the lowest relative abundance (1.5%), significantly lower than *P. nigromaculatus* (7.5%; adjusted *p* < 0.05), whereas *D. japonicus* (4.0%) and *P. chosenicus* (4.2%) showed intermediate values (adjusted *p* > 0.05). Verrucomicrobiota showed a significant overall difference among species (Kruskal–Wallis test: *χ* ^*2*^ = 10.473, *p* = 0.015). The mean relative abundance was higher in *P. nigromaculatus* (6.0%) and *P. chosenicus* (8.4%) than in *D. suweonensis* (2.7%) and *D. japonicus* (2.1%). However, after Holm–Bonferroni correction, no significant pairwise differences were detected between any species (adjusted *p* > 0.05).

At the genus level, the compositional differences were more pronounced. The two most abundant genera, *Verticiella* (*χ* ^2^ = 24.700, *p* < 0.001) and *Rhizobacter* (*χ* ^2^ = 24.750, *p* < 0.001), showed mutually exclusive patterns between genera (Figure 5B). *Verticiella* dominated the skin microbiota of *Dryophytes* species ( *D. japonicus* 21.9%, *D. suweonensis* 25.5%) but was virtually absent in *Pelophylax* species ( *P. nigromaculatus* 0.0%, *P. chosenicus* ~ 0.0%; adjusted *p* < 0.05), whereas *Rhizobacter* was strongly enriched in *Pelophylax* species ( *P. nigromaculatus* 9.3%, *P. chosenicus* 21.5%) and nearly absent in *Dryophytes* species ( *D. japonicus* 0.1%, *D. suweonensis* 0.2%; adjusted *p* < 0.05). No significant differences were detected among the genera (adjusted *p* > 0.05).

In addition to these dominant taxa, *D. suweonensis* was characterized by higher relative abundances of *Enhydrobacter* (10.3%; *χ* ^2^ = 20.082, *p* < 0.001) and *Deinococcus* (6.9%; *χ* ^2^ = 10.780, *p* = 0.013) compared with both *Pelophylax* species (adjusted *p* < 0.05), whereas *D. japonicus* showed intermediate values (adjusted *p* > 0.05). Conversely, *P. chosenicus* showed strong enrichment of *Comamonas* (9.4%; *χ* ^2^ = 14.812, *p* = 0.002) and *Luteolibacter* (6.3%; *χ* ^2^ = 20.876, *p* = 0.002), which were significantly higher than in *D. suweonensis* (adjusted *p* < 0.05). *P. nigromaculatus* showed the second highest abundance of *Luteolibacter* (1.6%), which was significantly higher than that of *D. suweonensis* (adjusted *p* < 0.05).

### 3.5 Microbial Network and Functional Prediction

**FIGURE 6:**
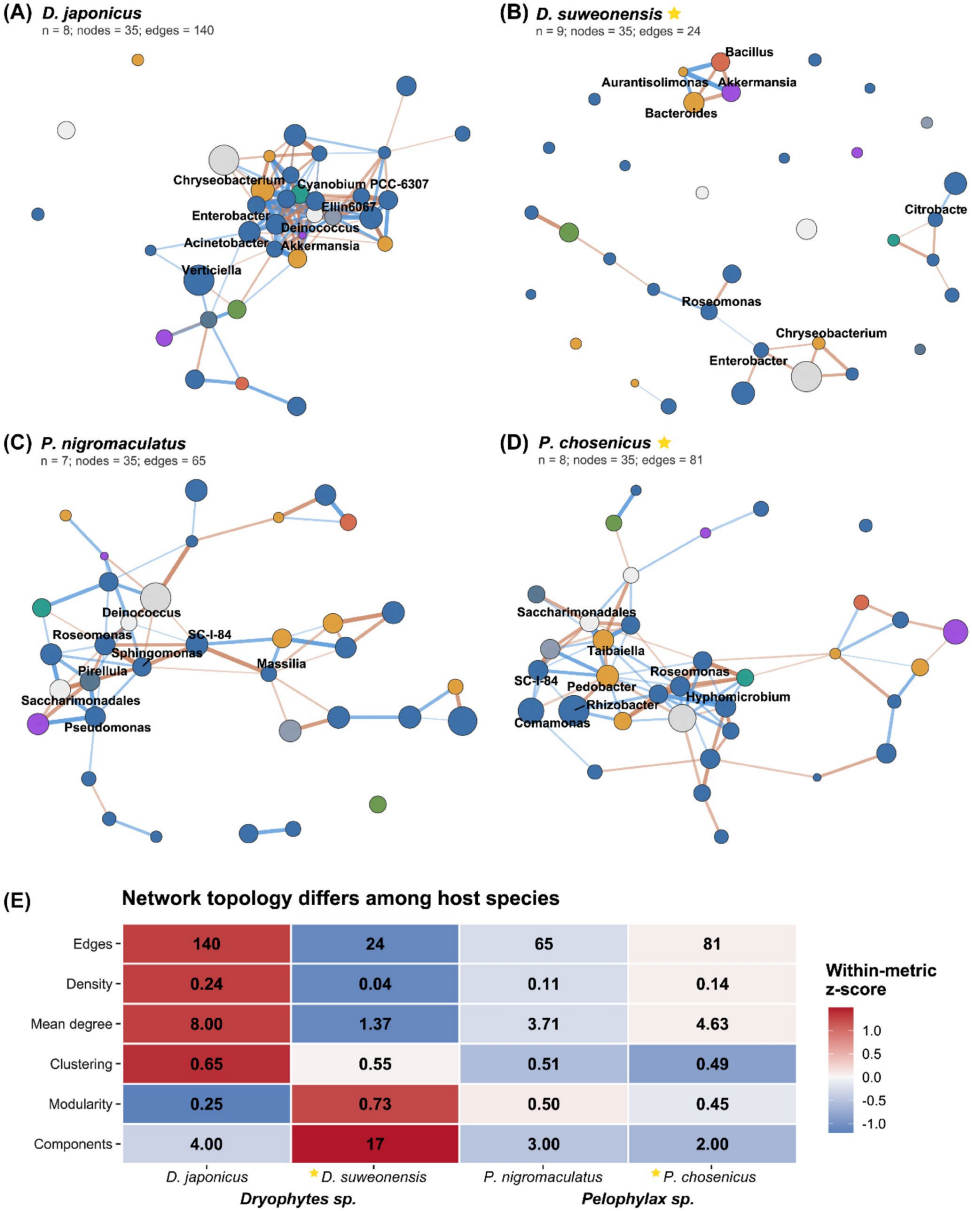
Species‐specific microbial co‐occurrence networks of skin bacterial communities across four frog species: (A) *Dryophytes japonicus* , (B) *D. suweonensis* , (C) *Pelophylax nigromaculatus* , and (D) *P. chosenicus* . Nodes represent bacterial taxa, and edges indicate significant co‐occurrence relationships. (E) Comparison of standardized network topological properties among species.

Species‐specific microbial co‐occurrence networks revealed marked differences in skin bacterial association structures among the four host species (Figure 6). All four networks were constructed with 35 bacterial nodes, but the number of retained associations differed substantially among species. The network of *D. japonicus* showed the highest connectivity, with 140 edges, a density of 0.235, and a mean degree of 8.00 (Figure 6A,E). In contrast, *D. suweonensis* showed the sparsest and most fragmented network, with only 24 edges, a density of 0.040, a mean degree of 1.37, and 17 connected components (Figure 6B,E). The two *Pelophylax* species showed intermediate network complexity. *P. nigromaculatus* had 65 edges, a density of 0.109, a mean degree of 3.71, and three connected components, whereas *P. chosenicus* had 81 edges, a density of 0.136, a mean degree of 4.63, and two connected components (Figure 6C,E). Modularity was highest in *D. suweonensis* (0.726), followed by *P. nigromaculatus* (0.498), *P. chosenicus* (0.450), and *D. japonicus* (0.247). These results indicate that skin bacterial association structures differed strongly among host species and did not follow a simple threatened‐versus‐common pattern.

**FIGURE 7:**
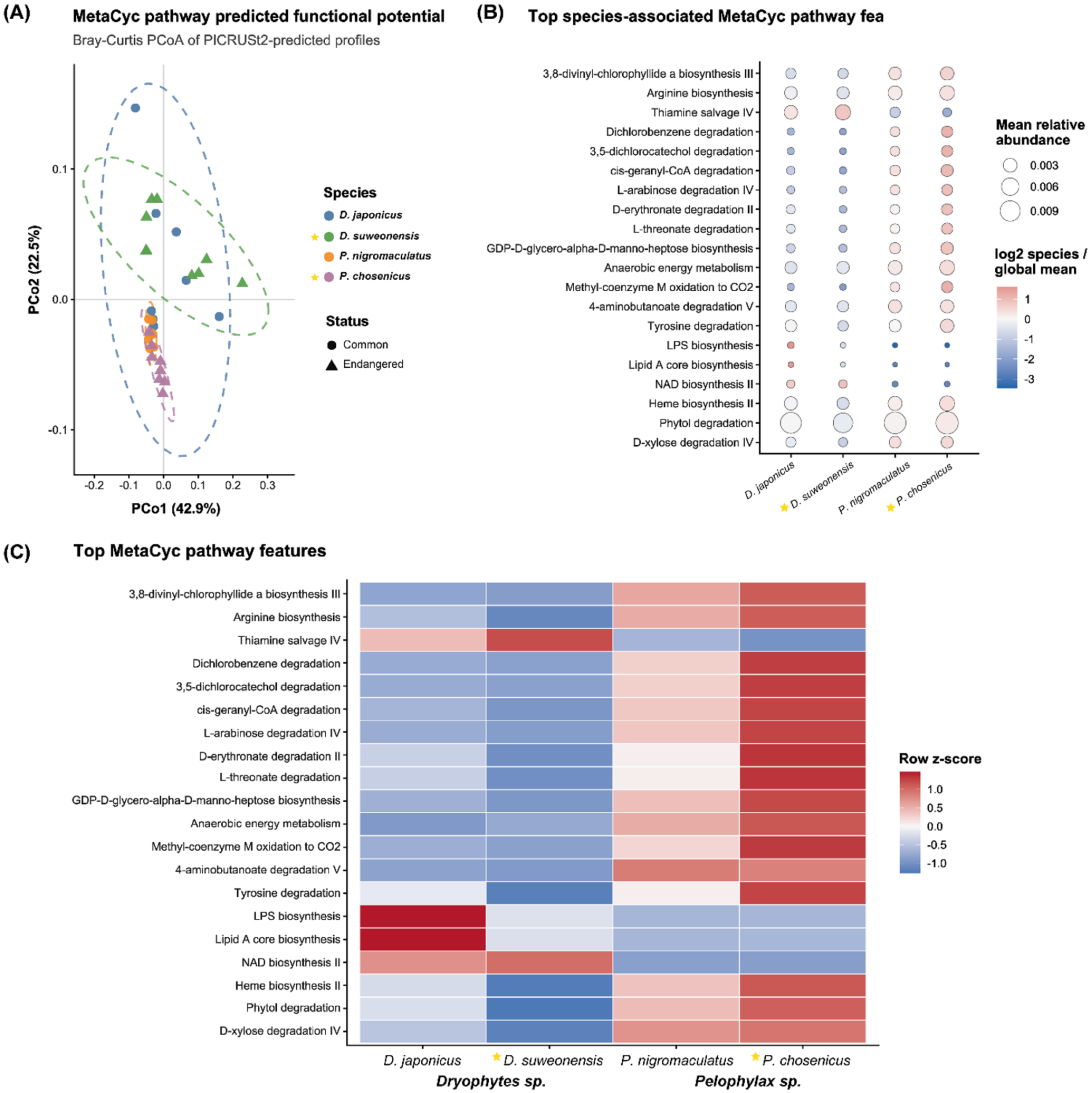
Predicted functional profiles of skin bacterial communities across four frog species based on PICRUSt2 and MetaCyc pathway inference: (A) principal coordinates analysis (PCoA) of Bray–Curtis dissimilarity, (B) bubble plot of top species‐associated pathway features, and (C) heatmap of top predicted pathway features.

PICRUSt2‐based functional prediction further showed that predicted functional potential was structured primarily by host species and host genus. Bray–Curtis PCoA of predicted MetaCyc pathway profiles showed partial separation among host species, with the first two axes explaining 42.9% and 22.5% of the variation, respectively (Figure 7A). PERMANOVA confirmed that predicted MetaCyc pathway composition differed significantly among species (*R* ^2^ = 0.278, *F* = 3.588, *p* = 0.001) and between host genera (*R* ^2^ = 0.192, *F* = 7.150, *p* = 0.001). In contrast, conservation status alone did not significantly explain predicted pathway composition (*R* ^2^ = 0.043, *F* = 1.332, *p* = 0.247). Feature‐level comparisons showed extensive species‐associated variation in predicted MetaCyc pathways, with 212 of 585 pathways remaining significant after false‐discovery‐rate correction at *q* < 0.05. The top species‐associated pathways included amino‐acid metabolism, cofactor biosynthesis, cell‐envelope‐related biosynthesis, and carbohydrate or aromatic‐compound degradation pathways (Figure 7B,C). Among the top 20 species‐associated pathways, 15 showed the highest mean abundance in *P. chosenicus* , whereas *D. suweonensis* showed the highest mean abundance for thiamine salvage IV and NAD biosynthesis II, and *D. japonicus* showed the highest mean abundance for LPS biosynthesis and lipid A core biosynthesis.

**FIGURE 8:**
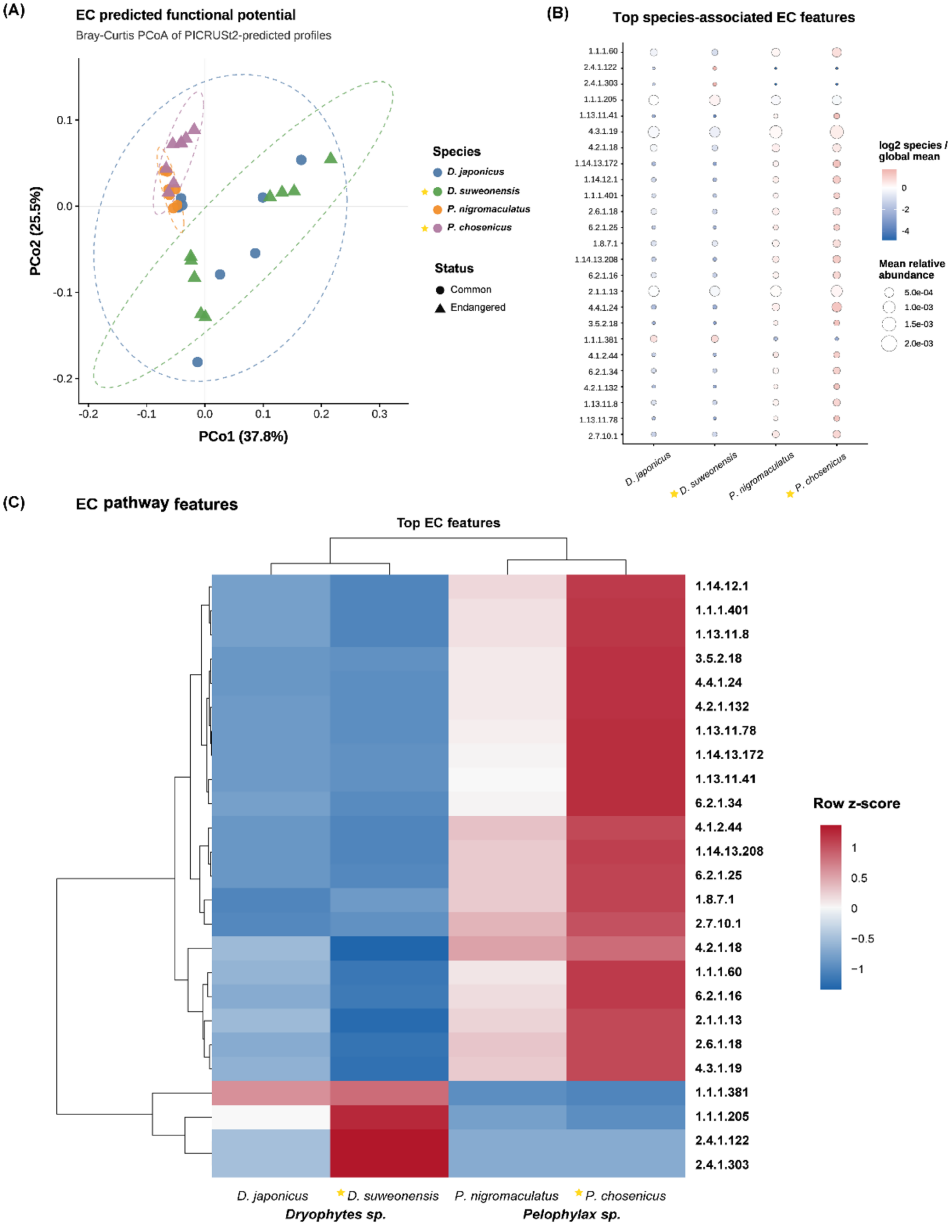
Predicted functional profiles of skin bacterial communities across four frog species based on PICRUSt2‐derived EC profiles. (A) Bray–Curtis PCoA of predicted EC profiles. (B) Top species‐associated EC features shown as a bubble plot. Bubble size indicates mean relative abundance and color indicates log^2^ enrichment relative to the global mean. (C) Heatmap of the top species‐associated EC features.

The EC‐based functional profiles showed a consistent pattern. EC‐level PCoA also showed species‐level structuring, with the first two axes explaining 37.8% and 25.5% of the variation, respectively (Figure 8). PERMANOVA indicated significant effects of species identity (*R* ^2^ = 0.326, *F* = 4.509, *p* = 0.001) and host genus (*R* ^2^ = 0.241, *F* = 9.528, *p* = 0.001), whereas conservation status alone was not significant (*R* ^2^ = 0.038, *F* = 1.201, *p* = 0.287). Together, the network and predicted functional analyzes support the interpretation that skin microbiome organization in these co‐occurring frogs is primarily structured by host species and genus‐level identity rather than by threatened status alone.

### 3.6 Integrative Analysis Using Redundancy Analysis

**FIGURE 9:**
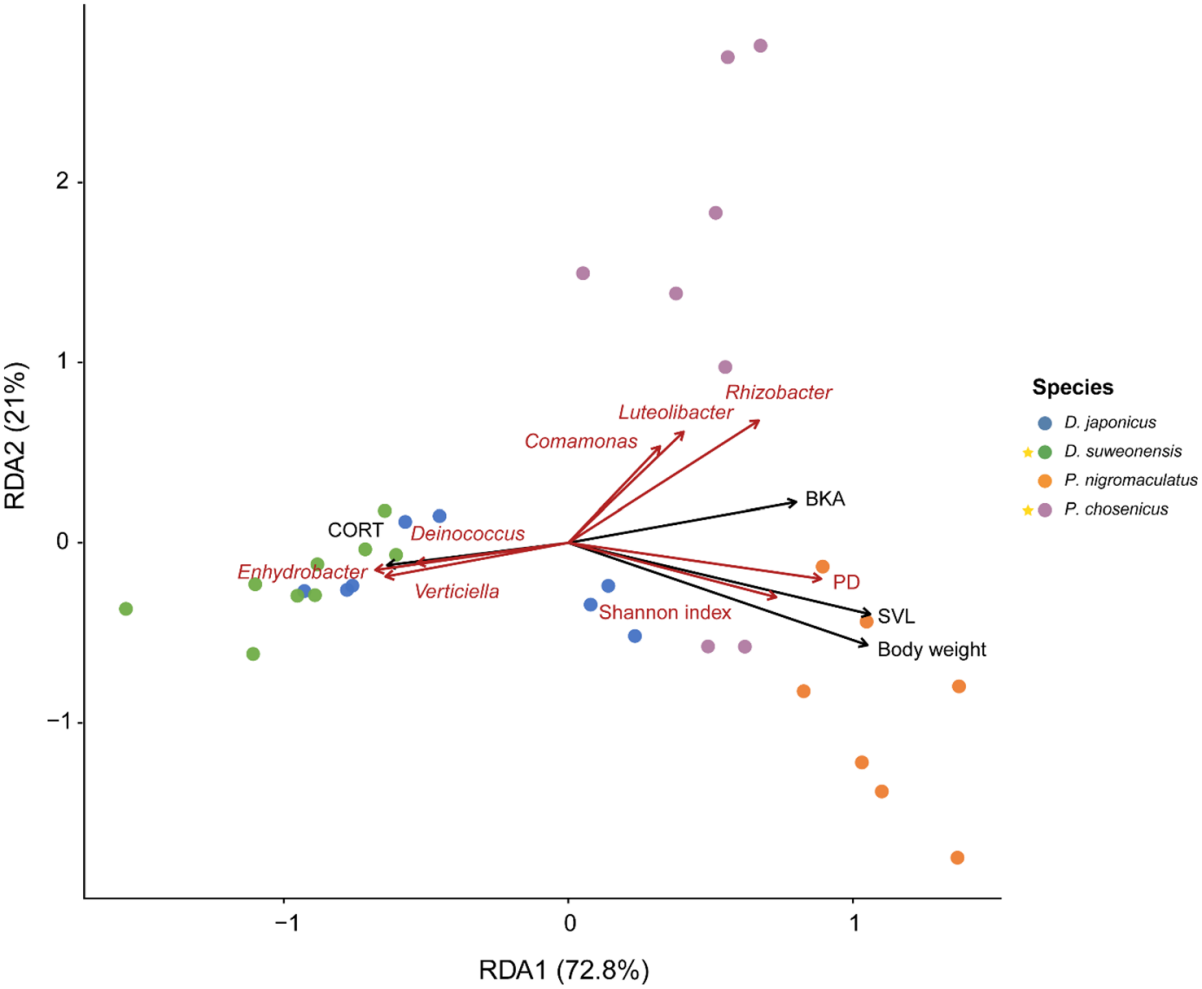
Redundancy analysis (RDA) of skin bacterial communities and host physiological variables across frog species.

RDA integrating physiological and microbiome variables revealed clear species‐level separation patterns (Figure 9). The constrained model explained 46.1% of the total variance (adjusted *R* ^2^ = 0.403) and was highly significant (*F* = 7.98, *p* = 0.001). The first two canonical axes accounted for 93.9% of the explained constrained variation. RDA1 explained 72.8% (eigenvalue = 4.03) of the variance and was strongly significant (*F* = 17.42, *p* = 0.001), whereas RDA2 explained an additional 21.0% (eigenvalue = 1.16) of the variance and was also significant (*F* = 5.03, *p* = 0.001). In contrast, RDA3 explained only 6.2% of the variation (eigenvalue = 0.34) and was not statistically significant (*F* = 1.47, *p* = 0.135).

Along RDA1, *P. nigromaculatus* and *P. chosenicus* were positioned in a positive direction, whereas *D. japonicus* and *D. suweonensis* were separated in opposite directions. *Dryophytes japonicus* showed a partial overlap with both *P. chosenicus* and *D. suweonensis* , reflecting its intermediate physiological and microbiome characteristics. Along RDA2, *P. chosenicus* was distributed in a positive direction, providing additional separation from the other three species, which were largely clustered toward the negative side.

Distinct associations between the explanatory variables and species clusters were evident (Table 1). Body size (SVL and body weight) and microbiome α‐diversity indices (Shannon index and phylogenetic diversity; PD) were aligned in the positive direction of RDA1 and the negative direction of RDA2, explaining the ordination pattern of *P. nigromaculatus* . *Dryophytes suweonensis* , and some *D. japonicus* individuals, were associated with microbial genera including *Verticiella*, *Deinococcus*, and *Enhydrobacter*, thereby projecting toward the negative side of RDA1 and aligning with elevated CORT levels. *P. chosenicus* showed strong associations with microbial taxa aligned in the positive direction of both RDA1 and RDA2, particularly *Comamonas*, *Luteolibacter*, and *Rhizobacter*. In addition, both *Pelophylax* species aligned with the BKA vector, reflecting their higher innate immune activity relative to the two *Dryophytes* species.

**TABLE 1:** Univariate and multivariate statistical analyzes of physiological traits and skin bacterial community structure across species.

Group	Variable	RDA1	RDA2	*R* ^2^	*p*
Physiology	Body weight	0.828	−0.369	0.822	0.001
Physiology	SVL	0.847	−0.31	0.814	0.001
Physiology	BKA	0.732	0.161	0.561	0.001
Physiology	CORT	−0.599	−0.084	0.365	0.004
Microbiome	Shannon index	0.773	−0.392	0.752	0.001
Microbiome	PD	0.847	−0.221	0.766	0.001
Microbiome	*Verticiella*	−0.593	−0.089	0.36	0.004
Microbiome	*Rhizobacter*	0.535	0.714	0.795	0.001
Microbiome	*Enhydrobacter*	−0.527	−0.117	0.292	0.008
Microbiome	*Comamonas*	0.305	0.532	0.377	0.003
Microbiome	*Deinococcus*	−0.451	−0.083	0.21	0.031
Microbiome	*Luteolibacter*	0.435	0.66	0.625	0.001

## 4 Discussion

### 4.1 Physiological Traits and Microbiome Diversity Among Species

In this study, we compared body size and mass, innate immunity, endocrine status, *Bd* infection, and skin bacterial diversity among four anuran species, including two endangered species and two closely related congeners, occurring in the same habitats. Overall, the four sympatric species differed in some physiological and microbiome‐related indices, with patterns structured primarily by taxonomic affiliation and species identity rather than conservation status alone. Several large‐scale comparative studies have reported that threatened species tend to harbor lower skin microbial diversity than that observed in nonthreatened species, even after accounting for major environmental and host‐related factors (Greenspan et al. 2022). In contrast, although common species tended to show higher skin bacterial richness and phylogenetic diversity than their threatened congeners, these trends were not statistically significant, indicating a partial overlap rather than categorical separation in baseline microbiome diversity. This discrepancy likely reflects the fine spatial scale and shared environmental context of our study, in which co‐occurring species were exposed to similar abiotic conditions and environmental microbial pools, potentially constraining divergence in baseline microbiome diversity and obscuring broad‐scale patterns detected across regions and biomes.

Recent biogeographic studies further suggest that both *D. suweonensis* and *P. chosenicus* , previously regarded as highly range‐restricted Korean species, may have broader distributions extending into northeastern China and adjacent regions of northeastern Asia (Borzée et al. 2024, 2026). Together, these findings suggest that the endangered status of these species in Korea may not necessarily be explained by strong baseline physiological or microbiome differences alone, as the endangered species examined in the present study exhibited broadly overlapping physiological and microbiome profiles relative to their widespread congeners. These results highlight the need for continued evaluation of conservation status in the context of regional population structure and local environmental conditions.

Extensive ecological studies have demonstrated that habitat configuration, connectivity, and water quality strongly influence the distribution and local persistence of both *D. suweonensis* and *P. chosenicus* in agricultural landscapes (Yang and Koo 2016; Borzée et al. 2018; Park et al. 2021). Habitat configuration and connectivity strongly impact habitat availability and population persistence in *D. suweonensis* , and continuous rice paddies with reduced fragmentation have been identified as the major predictors of its occurrence (Park et al. 2021). The *P. chosenicus* population has also been identified as declining because of habitat degradation. However, population increases have occurred in restored areas where wetland areas are expanded, overwintering sites are provided, movement pathways are secured, and drainage systems are improved. These results demonstrate that this species is highly sensitive to landscape‐level habitat conditions (Yang and Koo 2016). In addition, water quality influenced by fertilizers and other agricultural runoff was correlated with the occurrence patterns of both *D. suweonensis* and *P. chosenicus* , indicating that agricultural wetlands and their populations are closely linked (Borzée et al. 2018).

Beyond these well‐documented habitat‐level impacts, physiological and microbiological information can help specify how habitat‐level changes shape species responses (Cooke et al. 2013; Jiménez and Sommer 2017). Such information can help prioritize which factors are necessary to address when many broad drivers are extremely difficult to manage in field settings. Such prioritization is needed to determine the environmental conditions that are most relevant for sustaining local populations (Campbell Grant et al. 2020; Borzée et al. 2025). Taken together, our findings indicate that within shared agricultural wetland environments, sympatric anuran species exhibit broadly overlapping baseline physiological and microbiome profiles with subtle but structured differences among taxa. Rather than indicating clear intrinsic physiological or microbial disadvantages in endangered species, these results support the view that fine‐scale vulnerability and persistence may be associated with interactions between species traits and local habitat contexts. Accordingly, we suggest that physiological and microbiological indicators can serve as complementary tools for detecting how amphibian populations respond to habitat modification and management rather than as standalone predictors of population persistence.

### 4.2 Skin Microbiome Composition and Community Structure

The physiological patterns differed primarily at the genus level. The *Dryophytes* species ( *D. suweonensis* and *D. japonicus* ) were characterized by higher CORT concentrations, whereas the *Pelophylax* species ( *P. chosenicus* and *P. nigromaculatus* ) exhibited higher BKA levels. These physiological differences were consistently structured at the genus level rather than according to conservation status. Similar genus‐level differentiation was also observed in skin bacterial community composition, suggesting that closely related species occupying the same wetlands may nonetheless maintain distinct physiological and microbial profiles. Because all individuals were sampled during the breeding season, these patterns likely reflect stable intergeneric differences under shared environmental conditions rather than species‐specific responses associated with threatened status.

The bacterial community structure of the skin followed a similar pattern. Across species, *Dryophytes* were dominated by *Verticiella*, whereas *Pelophylax* was dominated by *Rhizobacter*. Rather than assigning specific functional roles to these taxa, these contrasting community profiles were best interpreted as signatures of genus‐level differentiation in host traits and environmental exposure within the same wetland system. The skin microbiome assembly in amphibians is strongly shaped by both host identity and environmental microbial pools (Muletz Wolz et al. 2018; Jani and Briggs 2018), and the observed genus‐specific dominance patterns likely reflect this joint filtering.

Several bacterial genera showed species‐specific enrichment only in endangered species. *Dryophytes suweonensis* exhibited high abundances of *Enhydrobacter* and *Deinococcus*. *Enhydrobacter* has been described in lake water (Staley et al. 1987), and *Deinococcus* is a well‐known stress‐tolerant bacterium that occurs on desiccated and UV‐exposed terrestrial surfaces (Mattimore and Battista 1996). These patterns may reflect the environmental exposure along the rice paddy edges used by *D. suweonensis* . Additionally, *P. chosenicus* showed higher abundances of *Comamonas* and *Luteolibacter*. Members of the family *Comamonadaceae*, including *Comamonas*, are frequently detected in nutrient‐rich freshwater and activated sludge environments (Gumaelius et al. 2001; Khan et al. 2002), and *Luteolibacter* is the dominant taxon in freshwater biofilms (Veach et al. 2016). Together, these species‐level enrichments suggest that closely related frogs occupying the same wetlands experience subtly different microbial landscapes, which are potentially linked to microhabitat use, behavior, or skin properties. Whether these differences translate into functional consequences for host defense or physiological regulation remains an important topic for future research (Woodhams et al. 2014; Rebollar et al. 2020).

The network and functional prediction analyzes further supported this interpretation. Species‐specific co‐occurrence networks differed markedly in connectivity and fragmentation, with *D. japonicus* showing the densest association structure and *D. suweonensis* showing the sparsest and most fragmented network. However, this pattern was not mirrored by conservation status alone because *P. chosenicus* , despite being endangered, retained a more connected network than *D. suweonensis* . These results suggest that skin microbiome organization is not simply reduced or destabilized in endangered species, but instead reflects host species identity, genus‐level traits, and potentially species‐specific microhabitat exposure. Because co‐occurrence networks are based on statistical associations, these patterns should be interpreted as differences in community association structure rather than direct microbial interactions (Röttjers and Faust 2018).

The PICRUSt2‐based functional prediction showed a similar pattern. Predicted MetaCyc pathway and EC profiles were significantly structured by host species and host genus, whereas threatened status alone explained little variation. Thus, the functional prediction results were consistent with the taxonomic and network analyzes in indicating host‐identity‐associated differentiation rather than a general functional deficit in endangered species. The species‐associated predicted pathways included amino‐acid metabolism, cofactor biosynthesis, cell‐envelope‐related biosynthesis, and carbohydrate or aromatic‐compound degradation pathways. These patterns may reflect differences in microbial resource environments or host‐associated filtering processes, but they should be interpreted conservatively because PICRUSt2 infers functional potential from 16S rRNA gene data rather than directly measuring metagenomic or transcriptomic activity (Douglas et al. 2020).

Notably, neither *Bd* prevalence nor *Bd* infection intensity differed among species despite clear differences in skin bacterial community composition. Although amphibian skin microbiota are widely recognized as important components of host defense against *Bd* (Bletz et al. 2017; Rebollar et al. 2020), the distinct microbial assemblages observed among sympatric species were not accompanied by corresponding differences in current *Bd* infection patterns. A large proportion of East Asian amphibians are known to maintain persistent *Bd* infections without severe population declines (O'Hanlon et al. 2018; Fu and Waldman 2019), which may explain why marked interspecific differences in *Bd* infection intensity were not observed in the present study. These findings suggest that baseline variation in skin bacterial composition alone may not directly predict *Bd* infection outcomes under shared environmental conditions.

### 4.3 Integrated Patterns and Conservation Context

In the PCoA based on physiological indicators, *D. japonicus* and *D. suweonensis* showed a strong overlap, whereas *P. nigromaculatus* and *P. chosenicus* were more clearly separated. This pattern was informative at the community level. The partial separation between the two *Pelophylax* species suggests that they may be influenced by different environmental factors or may respond differently to similar conditions. Therefore, it is important to determine whether this separation corresponds to ecologically distinct or complementary roles within local communities. Differences in environmental responsiveness among species may contribute to the maintenance of community‐level diversity, suggesting that conservation efforts within a single habitat may need to prioritize *P. chosenicus* rather than treat both *Pelophylax* species equally. In contrast, the strong overlap between the two *Dryophytes* species indicates highly similar physiological profiles, implying that environmental pressures acting on one species are likely to affect the other in parallel. Accordingly, management strategies should consider *D. suweonensis* and *D. japonicus* together with particular attention to shared habitat‐level pressures.

These multivariate patterns emphasize that sympatric assemblages are not functionally uniform even when species occupy the same wetlands and share similar life histories. Instead, fine‐scale physiological structuring among congeners may represent different adaptive strategies or sensitivities that shape the propagation of environmental stressors through local communities (Muletz Wolz et al. 2018; Hernández‐Gómez and Hua 2023). Based on empirical field observations, some habitats harbored a greater number of endangered individuals than common congeners, and only endangered species were present at certain sites. Comparative analyzes across such habitats may provide further insights into the environmental and ecological conditions that allow persistence. For example, *D. japonicus* showed intermediate physiological and skin bacterial characteristics and overlapped with all four focal species. The presence of such species raises questions about whether skin‐associated bacteria or other ecological interactions may be horizontally shared among co‐occurring species and whether this overlap contributes to persistence or buffering effects for endangered species. Evaluating whether this functional overlap facilitates coexistence, modulates disease risk, or buffers populations against disturbances is a promising direction for community‐level conservation physiology (Rebollar et al. 2020; Hernández‐Gómez and Hua 2023).

However, several limitations of this study should be considered when interpreting the results. Sample sizes for each species were relatively small because of the ethical and permitting constraints associated with sampling endangered amphibians. Our analyzes were conducted within a single geographic region, focusing on a specific habitat type and sampling period, and all four species were sampled from the same agricultural wetland landscapes during comparable life‐history stages. Therefore, the absence of significant differences in physiological and microbiome traits may partly reflect the fact that these species occur in a shared environmental context. Comparative analyzes across a broader range of habitats, particularly those where endangered species are dominant or occur exclusively, could provide additional insights into the conditions that promote persistence. Expanding analyzes across seasons and multiple developmental stages is also important to understand how physiological and microbial communities shift from the individual to the assemblage level within agricultural wetland ecosystems.

Such extensions are essential for determining how the fine‐scale functional divergence among sympatric species scales up to population trajectories and community stability under environmental changes. Such work, especially if it incorporates functional perspectives on microbial taxa and host traits, would provide a more robust framework for amphibian conservation from the population to the community scale and reinforce the need to interpret amphibian decline in terms of both species‐ and community‐level traits.

## Author Contributions

**Ji‐Eun Lee:** conceptualization (equal), data curation (equal), formal analysis (equal), investigation (equal), visualization (equal), writing – original draft (equal). **Jun‐Sung Kim:** data curation (equal), formal analysis (equal), investigation (equal). **Yuno Do:** methodology (equal), project administration (equal), resources (equal), software (equal), supervision (equal), writing – review and editing (equal). **Jun‐Kyu Park:** conceptualization (equal), funding acquisition (equal), investigation (equal), methodology (equal), software (equal), validation (equal), writing – original draft (equal).

## Funding

This work was supported by the National Research Foundation of Korea (Grants RS‐2024‐00349377 and RS‐2026‐2547469030782064780001).

## Conflicts of Interest

The authors declare no conflicts of interest.

## Supporting information


**Data S1:** Supplementary_Table_Functional.

## Data Availability

The data and code supporting this study are available from the Dryad Digital Repository: https://doi.org/10.5061/dryad.ns1rn8q7p (https://datadryad.org/share/GuthudC5T8Ul2gVqLb9WHgsFak2HSDI6K‐9Hop‐Mtto). The archive includes raw Illumina MiSeq FASTQ files, processed QIIME 2 output files, sample metadata, physiological and Bd infection data, and R and shell scripts used for physiological, microbiome diversity, taxonomic composition, PICRUSt2‐based functional prediction, and microbial co‐occurrence network analyzes.

## References

[ece373944-bib-0001] Assis, V. R. , S. C. M. Titon , A. M. G. Barsotti , B. Spira , and F. R. Gomes . 2013. “Antimicrobial Capacity of Plasma From Anurans of the Atlantic Forest.” South American Journal of Herpetology 8, no. 3: 155–160.

[ece373944-bib-0002] Assis, V. R. , S. C. M. Titon , and F. R. Gomes . 2019. “Acute Stress, Steroid Plasma Levels, and Innate Immunity in Brazilian Toads.” General and Comparative Endocrinology 273: 86–97. 29750968 10.1016/j.ygcen.2018.05.008

[ece373944-bib-0003] Beranek, C. T. , A. J. Hamer , S. V. Mahony , et al. 2023. “Severe Wildfires Promoted by Climate Change Negatively Impact Forest Amphibian Metacommunities.” Diversity and Distributions 29, no. 6: 785–800.

[ece373944-bib-0004] Blaustein, A. R. , S. S. Gervasi , P. T. J. Johnson , et al. 2012. “Ecophysiology Meets Conservation: Understanding the Role of Disease in Amphibian Population Declines.” Philosophical Transactions of the Royal Society of London. Series B, Biological Sciences 367, no. 1596: 1688–1707. 22566676 10.1098/rstb.2012.0011 PMC3350657

[ece373944-bib-0005] Blaustein, A. R. , and J. M. Kiesecker . 2002. “Complexity in Conservation: Lessons From the Global Decline of Amphibian Populations.” Ecology Letters 5, no. 4: 597–608.

[ece373944-bib-0201] Bletz, M. C. , J. Myers , D. C. Woodhams , et al. 2017. “Estimating herd immunity to amphibian chytridiomycosis in Madagascar based on the defensive function of amphibian skin bacteria.” Frontiers in Microbiology 8: 1751. 28959244 10.3389/fmicb.2017.01751 PMC5604057

[ece373944-bib-0006] Bolyen, E. , J. R. Rideout , M. R. Dillon , et al. 2019. “Reproducible, Interactive, Scalable and Extensible Microbiome Data Science Using QIIME 2.” Nature Biotechnology 37, no. 8: 852–857. 10.1038/s41587-019-0209-9 PMC7015180 31341288

[ece373944-bib-0007] Borzée, A. , C. N. Kyong , H. K. Kil , and Y. Jang . 2018. “Impact of Water Quality on the Occurrence of Two Endangered Korean Anurans: *Dryophytes suweonensis* and *Pelophylax chosenicus* .” Herpetologica 74: 1–7.

[ece373944-bib-0008] Borzée, A. , V. K. Prasad , K. Neam , et al. 2025. “Conservation Priorities for Global Amphibian Biodiversity.” Nature Reviews Biodiversity 1, no. 12: 1–18.

[ece373944-bib-0009] Borzée, A. , Y. Shin , Y. Bae , et al. 2024. “From Korean to Northeast Asian Endemicity: On the Occurrence of *Pelophylax chosenicus* Along the Eastern Coastal Yellow Sea.” Frontiers of Biogeography 16, no. 2: 1–9.

[ece373944-bib-0010] Borzée, A. , T. E. Um , V. K. Prasad , A. Shrivastava , M. S. Min , and S. N. Othman . 2026. “From Threatened to Widespread: A Non‐Genuine Conservation Status Update for a Now Widespread Asian Hylid.” Animal Cells and Systems 30, no. 1: 115–130. 41727212 10.1080/19768354.2026.2614151 PMC12918279

[ece373944-bib-0011] Boyle, D. G. , D. B. Boyle , V. Olsen , J. A. Morgan , and A. D. Hyatt . 2004. “Rapid Quantitative Detection of Chytridiomycosis (*Batrachochytrium dendrobatidis*) in Amphibian Samples Using Real‐Time TaqMan PCR Assay.” Diseases of Aquatic Organisms 60, no. 2: 141–148. 15460858 10.3354/dao060141

[ece373944-bib-0013] Callahan, B. J. , P. J. McMurdie , M. J. Rosen , A. W. Han , A. J. A. Johnson , and S. P. Holmes . 2016. “DADA2: High‐Resolution Sample Inference From Illumina Amplicon Data.” Nature Methods 13, no. 7: 581–583. 27214047 10.1038/nmeth.3869 PMC4927377

[ece373944-bib-0014] Campbell Grant, E. H. , D. A. W. Miller , and E. Muths . 2020. “A Synthesis of Evidence of Drivers of Amphibian Declines.” Herpetologica 76, no. 2: 101–107.

[ece373944-bib-0015] Caspi, R. , R. Billington , I. M. Keseler , et al. 2020. “The MetaCyc Database of Metabolic Pathways and Enzymes—A 2019 Update.” Nucleic Acids Research 48, no. D1: D445–D453. 31586394 10.1093/nar/gkz862 PMC6943030

[ece373944-bib-0016] Catenazzi, A. , E. Lehr , and V. T. Vredenburg . 2014. “Thermal Physiology, Disease, and Amphibian Declines on the Eastern Slopes of the Andes.” Conservation Biology: The Journal of the Society for Conservation Biology 28, no. 2: 509–517. 24372791 10.1111/cobi.12194

[ece373944-bib-0017] Cooke, S. J. , L. Sack , C. E. Franklin , et al. 2013. “What Is Conservation Physiology? Perspectives on an Increasingly Integrated and Essential Science (†).” Conservation Physiology 1, no. 1: cot001. 27293585 10.1093/conphys/cot001 PMC4732437

[ece373944-bib-0018] Douglas, G. M. , V. J. Maffei , J. R. Zaneveld , et al. 2020. “PICRUSt2 for Prediction of Metagenome Functions.” Nature Biotechnology 38, no. 6: 685–688. 10.1038/s41587-020-0548-6 PMC7365738 32483366

[ece373944-bib-0019] Edwards, O. M. , L. Zhai , M. S. Reichert , C. A. Shaughnessy , L. Ozment , and B. Zhang . 2025. “Physiological and Morphological Traits Affect Contemporary Range Expansion and Implications for Species Distribution Modelling in an Amphibian Species.” Journal of Animal Ecology 94, no. 2: 195–209. 39462865 10.1111/1365-2656.14212

[ece373944-bib-0020] Falaschi, M. , R. Manenti , W. Thuiller , and G. F. Ficetola . 2019. “Continental‐Scale Determinants of Population Trends in European Amphibians and Reptiles.” Global Change Biology 25, no. 10: 3504–3515. 31220393 10.1111/gcb.14739

[ece373944-bib-0021] Fontaine, S. S. , and K. D. Kohl . 2023. “The Microbiome Buffers Tadpole Hosts From Heat Stress: A Hologenomic Approach to Understand Host–Microbe Interactions Under Warming.” Journal of Experimental Biology 226, no. 1: jeb245191. 36546449 10.1242/jeb.245191 PMC10086385

[ece373944-bib-0022] Forzán, M. J. , R. V. Vanderstichel , C. T. Ogbuah , J. R. Barta , and T. G. Smith . 2012. “Blood Collection From the Facial (Maxillary)/Musculo‐Cutaneous Vein in True Frogs (Family Ranidae).” Journal of Wildlife Diseases 48, no. 1: 176–180. 22247387 10.7589/0090-3558-48.1.176

[ece373944-bib-0023] Fu, M. , and B. Waldman . 2019. “Ancestral Chytrid Pathogen Remains Hypervirulent Following Its Long Coevolution With Amphibian Hosts.” Proceedings of the Royal Society B: Biological Sciences 286: 20190833. 10.1098/rspb.2019.0833 PMC6571470 31161901

[ece373944-bib-0024] Gallagher, R. V. , N. Butt , A. J. R. Carthey , et al. 2021. “A Guide to Using Species Trait Data in Conservation.” One Earth 4, no. 7: 927–936.

[ece373944-bib-0025] Grant, E. H. C. , D. A. W. Miller , B. R. Schmidt , et al. 2016. “Quantitative Evidence for the Effects of Multiple Drivers on Continental‐Scale Amphibian Declines.” Scientific Reports 6: 25625. 27212145 10.1038/srep25625 PMC4876446

[ece373944-bib-0026] Greenspan, S. E. , P. Peloso , J. A. Fuentes‐González , et al. 2022. “Low Microbiome Diversity in Threatened Amphibians From Two Biodiversity Hotspots.” Animal Microbiome 4, no. 1: 69. 36582011 10.1186/s42523-022-00220-w PMC9801548

[ece373944-bib-0027] Gumaelius, L. , G. Magnusson , B. Pettersson , and G. Dalhammar . 2001. “ *Comamonas denitrificans* sp. Nov., an Efficient Denitrifying Bacterium Isolated From Activated Sludge.” International Journal of Systematic and Evolutionary Microbiology 51, no. 3: 999–1006. 11411726 10.1099/00207713-51-3-999

[ece373944-bib-0028] Hammond, T. T. , Z. A. Au , A. C. Hartman , and C. L. Richards‐Zawacki . 2018. “Assay Validation and Interspecific Comparison of Salivary Glucocorticoids in Three Amphibian Species.” Conservation Physiology 6, no. 1: coy055. 30279992 10.1093/conphys/coy055 PMC6158758

[ece373944-bib-0030] Hernández‐Gómez, O. , and J. Hua . 2023. “From the Organismal to Biosphere Levels: Environmental Impacts on the Amphibian Microbiota.” FEMS Microbiology Reviews 47, no. 1: fuad002. 36725211 10.1093/femsre/fuad002

[ece373944-bib-0031] Hof, C. , M. B. Araújo , W. Jetz , and C. Rahbek . 2011. “Additive Threats From Pathogens, Climate and Land‐Use Change for Global Amphibian Diversity.” Nature 480, no. 7378: 516–519. 22089134 10.1038/nature10650

[ece373944-bib-0032] Hou, K. , Z.‐X. Wu , X.‐Y. Chen , et al. 2022. “Microbiota in Health and Diseases.” Signal Transduction and Targeted Therapy 7, no. 1: 135. 35461318 10.1038/s41392-022-00974-4 PMC9034083

[ece373944-bib-0033] Hyatt, A. 2017. Infection With Batrachochytrium dendrobatidis Manual of Diagnostic Tests for Aquatic Animals. World Organization for Animal Health (OIE).

[ece373944-bib-0034] Hyatt, A. D. , D. G. Boyle , V. Olsen , et al. 2007. “Diagnostic Assays and Sampling Protocols for the Detection of *Batrachochytrium dendrobatidis* .” Diseases of Aquatic Organisms 73, no. 3: 175–192. 17330737 10.3354/dao073175

[ece373944-bib-0035] Illumina . 2011. Preparing 16S Ribosomal RNA Gene Amplicons for the Illumina MiSeq System. Illumina Technical Note. https://www.illumina.com/content/dam/illumina‐support/documents/documentation/chemistry_documentation/16s/16s‐metagenomic‐library‐prep‐guide‐15044223‐b.pdf.

[ece373944-bib-0037] Jani, A. J. , and C. J. Briggs . 2018. “Host and Aquatic Environment Shape the Amphibian Skin Microbiome but Effects on Downstream Resistance to the Pathogen *Batrachochytrium dendrobatidis* Are Variable.” Frontiers in Microbiology 9: 487. 29619014 10.3389/fmicb.2018.00487 PMC5871691

[ece373944-bib-0039] Jiménez, R. R. , and S. Sommer . 2017. “The Amphibian Microbiome: Natural Range of Variation, Pathogenic Dysbiosis, and Role in Conservation.” Biodiversity and Conservation 26, no. 4: 763–786.

[ece373944-bib-0040] Khan, S. T. , Y. Horiba , M. Yamamoto , and A. Hiraishi . 2002. “Members of the Family *Comamonadaceae* as Primary Poly(3‐Hydroxybutyrate‐Co‐3‐Hydroxyvalerate)‐Degrading Denitrifiers in Activated Sludge as Revealed by a Polyphasic Approach.” Applied and Environmental Microbiology 68, no. 7: 3206–3214. 12088996 10.1128/AEM.68.7.3206-3214.2002 PMC126756

[ece373944-bib-0041] Knapp, R. A. , G. M. Fellers , P. M. Kleeman , et al. 2016. “Large‐Scale Recovery of an Endangered Amphibian Despite Ongoing Exposure to Multiple Stressors.” Proceedings of the National Academy of Sciences of the United States of America 113, no. 42: 11889–11894. 27698128 10.1073/pnas.1600983113 PMC5081604

[ece373944-bib-0042] Kueneman, J. G. , M. C. Bletz , V. J. McKenzie , et al. 2019. “Community Richness of Amphibian Skin Bacteria Correlates With Bioclimate at the Global Scale.” Nature Ecology & Evolution 3, no. 3: 381–389. 30778181 10.1038/s41559-019-0798-1

[ece373944-bib-0043] Lee, J.‐E. , J.‐K. Park , and Y. Do . 2024. “Gut Microbiome Diversity and Function During Hibernation and Spring Emergence in an Aquatic Frog.” PLoS One 19, no. 2: e0298245. 38363754 10.1371/journal.pone.0298245 PMC10871480

[ece373944-bib-0044] Lee, J.‐E. , J.‐K. Park , and Y. Do . 2025. “Analysis of Reproductive Strategies and Immunological Interactions in *Batrachochytrium dendrobatidis*‐Resistant Japanese Tree Frogs.” Animals: An Open Access Journal From MDPI 15, no. 2: 154. 39858154 10.3390/ani15020154 PMC11758651

[ece373944-bib-0045] Levin, S. A. 1992. “The Problem of Pattern and Scale in Ecology: The Robert H. MacArthur Award Lecture.” Ecology 73, no. 6: 1943–1967.

[ece373944-bib-0046] Liu, C. , Y. Cui , X. Li , and M. Yao . 2021. “ *Microeco*: An R Package for Data Mining in Microbial Community Ecology.” FEMS Microbiology Ecology 97, no. 2: fiaa255. 33332530 10.1093/femsec/fiaa255

[ece373944-bib-0047] Liu, G. , J. J. L. Rowley , R. T. Kingsford , and C. T. Callaghan . 2021. “Species' Traits Drive Amphibian Tolerance to Anthropogenic Habitat Modification.” Global Change Biology 27, no. 13: 3120–3132. 33939215 10.1111/gcb.15623

[ece373944-bib-0048] Luedtke, J. A. , J. Chanson , K. Neam , et al. 2023. “Ongoing Declines for the World's Amphibians in the Face of Emerging Threats.” Nature 622, no. 7982: 308–314. 37794184 10.1038/s41586-023-06578-4 PMC10567568

[ece373944-bib-0049] Lynch, J. B. , and E. Y. Hsiao . 2019. “Microbiomes as Sources of Emergent Host Phenotypes.” Science 365, no. 6460: 1405–1409. 31604267 10.1126/science.aay0240

[ece373944-bib-0050] Madison, J. D. , O. G. Osborne , A. Ellison , et al. 2025. “Probiotic Colonization of *Xenopus laevis* Skin Causes Short‐Term Changes in Skin Microbiomes and Gene Expression.” Infection and Immunity 93: e00569‐24. 40172536 10.1128/iai.00569-24 PMC12070741

[ece373944-bib-0051] Mattimore, V. , and J. R. Battista . 1996. “Radioresistance of *Deinococcus radiodurans* : Functions Necessary to Survive Ionizing Radiation Are Also Necessary to Survive Prolonged Desiccation.” Journal of Bacteriology 178, no. 3: 633–637. 8550493 10.1128/jb.178.3.633-637.1996 PMC177705

[ece373944-bib-0052] Muletz Wolz, C. R. , S. A. Yarwood , E. H. C. Grant , R. C. Fleischer , and K. R. Lips . 2018. “Effects of Host Species and Environment on the Skin Microbiome of Co‐Occurring Amphibians.” Journal of Animal Ecology 87: 1426–1438. 10.1111/1365-2656.12726 28682480

[ece373944-bib-0053] Neely, W. J. , R. A. Martins , C. M. Mendonça da Silva , et al. 2023. “Linking Microbiome and Stress Hormone Responses in Wild Tropical Treefrogs Across Continuous and Fragmented Forests.” Communications Biology 6, no. 1: 1261. 38087051 10.1038/s42003-023-05600-9 PMC10716138

[ece373944-bib-0202] O'Hanlon, S. J. , A. Rieux , R. A. Farrer , et al. 2018. “Recent Asian origin of chytrid fungi causing global amphibian declines.” Science 360, no. 6389: 621–627. 29748278 10.1126/science.aar1965 PMC6311102

[ece373944-bib-0054] Oksanen, J. , F. G. Blanchet , R. Kindt , et al. 2013. “Package ‘Vegan’. Community Ecology Package, Version 2:1–295.”

[ece373944-bib-0055] Park, I.‐K. , D. Park , and A. Borzée . 2021. “Defining Conservation Requirements for the Suweon Treefrog ( *Dryophytes suweonensis* ) Using Species Distribution Models.” Diversity 13, no. 2: 69.

[ece373944-bib-0056] Park, J.‐K. , and Y. Do . 2022. “Wind Turbine Noise Behaviorally and Physiologically Changes Male Frogs.” Biology 11, no. 4: 516. 35453715 10.3390/biology11040516 PMC9031316

[ece373944-bib-0057] Park, J.‐K. , and Y. Do . 2023. “Current State of Conservation Physiology for Amphibians: Major Research Topics and Physiological Parameters.” Animals: An Open Access Journal From MDPI 13, no. 20: 3162. 37893886 10.3390/ani13203162 PMC10603670

[ece373944-bib-0058] Park, J.‐K. , and Y. Do . 2024. “Combined Effect of Seasons and Life History in an Anuran Strengthens the Response and Relationship Between Their Physiology and Gut Microbiota.” Scientific Reports 14, no. 1: 10137. 38698108 10.1038/s41598-024-60105-7 PMC11066060

[ece373944-bib-0059] Park, J.‐K. , J. E. Lee , and Y. Do . 2025. “Leveraging Multi‐Level Biomarkers Using Machine Learning: Identifying Physiological and Skin Microbial Dynamics in *Bd*‐Resistant Amphibians.” Integrative Zoology 21: 624–638. 40689719 10.1111/1749-4877.13015

[ece373944-bib-0060] Pruesse, E. , C. Quast , K. Knittel , et al. 2007. “SILVA: A Comprehensive Online Resource for Quality Checked and Aligned Ribosomal RNA Sequence Data Compatible With ARB.” Nucleic Acids Research 35, no. 21: 7188–7196. 17947321 10.1093/nar/gkm864 PMC2175337

[ece373944-bib-0061] Rebollar, E. A. , E. Martínez‐Ugalde , and A. H. Orta . 2020. “The Amphibian Skin Microbiome and Its Protective Role Against Chytridiomycosis.” Herpetologica 76, no. 2: 167–177.

[ece373944-bib-0062] Richardson, J. M. L. 2002. “A Comparative Study of Phenotypic Traits Related to Resource Utilization in Anuran Communities.” Evolutionary Ecology 16, no. 2: 101–122.

[ece373944-bib-0063] Rohr, J. R. , A. M. Schotthoefer , T. R. Raffel , et al. 2008. “Agrochemicals Increase Trematode Infections in a Declining Amphibian Species.” Nature 455, no. 7217: 1235–1239. 18972018 10.1038/nature07281

[ece373944-bib-0064] Rollins‐Smith, L. A. 2017. “Amphibian Immunity–Stress, Disease, and Climate Change.” Developmental & Comparative Immunology 66: 111–119. 27387153 10.1016/j.dci.2016.07.002

[ece373944-bib-0065] Romero, L. M. , and J. M. Reed . 2005. “Collecting Baseline Corticosterone Samples in the Field: Is Under 3 Min Good Enough?” Comparative Biochemistry and Physiology. Part A, Molecular & Integrative Physiology 140, no. 1: 73–79. 10.1016/j.cbpb.2004.11.004 15664315

[ece373944-bib-0066] Röttjers, L. , and K. Faust . 2018. “From Hairballs to Hypotheses—Biological Insights From Microbial Networks.” Nature Reviews Microbiology 16, no. 12: 731–740. 30085090 10.1093/femsre/fuy030 PMC6199531

[ece373944-bib-0067] Soberón, J. , and B. Arroyo‐Peña . 2017. “Are Fundamental Niches Larger Than the Realized? Testing a 50‐Year‐Old Prediction by Hutchinson.” PLoS One 12, no. 4: e0175138. 28403170 10.1371/journal.pone.0175138 PMC5389801

[ece373944-bib-0068] Staley, J. T. , R. L. Irgens , and D. J. Brenner . 1987. “ *Enhydrobacter aerosaccus* Gen. Nov., sp. Nov., a Gas‐Vacuolated, Facultatively Anaerobic, Heterotrophic Rod.” International Journal of Systematic Bacteriology 37, no. 3: 289–291.

[ece373944-bib-0069] Stuart, S. N. , J. S. Chanson , N. A. Cox , et al. 2004. “Status and Trends of Amphibian Declines and Extinctions Worldwide.” Science 306, no. 5702: 1783–1786. 15486254 10.1126/science.1103538

[ece373944-bib-0070] Team, R. C . 2016. R: A Language and Environment for Statistical Computing. R Foundation for Statistical Computing. http://www.R‐project.org/.

[ece373944-bib-0071] Titon, S. C. M. , B. Titon Junior , V. R. Assis , et al. 2020. “Hormonal Daily Variation Co‐Varies With Immunity in Captive Male Bullfrogs ( *Lithobates catesbeianus* ).” General and Comparative Endocrinology 303: 113702. 33359060 10.1016/j.ygcen.2020.113702

[ece373944-bib-0072] Veach, A. M. , J. C. Stegen , S. P. Brown , W. K. Dodds , and A. Jumpponen . 2016. “Spatial and Successional Dynamics of Microbial Biofilm Communities in a Grassland Stream Ecosystem.” Molecular Ecology 25, no. 18: 4674–4688. 27481285 10.1111/mec.13784

[ece373944-bib-0073] Walls, S. C. , and C. R. Gabor . 2019. “Integrating Behavior and Physiology Into Strategies for Amphibian Conservation.” Frontiers in Ecology and Evolution 7: 234.

[ece373944-bib-0074] Wiens, J. A. 1989. “Spatial Scaling in Ecology.” Functional Ecology 3, no. 4: 385–397.

[ece373944-bib-0075] Woodhams, D. C. , R. A. Alford , C. J. Briggs , M. Johnson , and L. A. Rollins‐Smith . 2014. “Life‐History Trade‐Offs Influence Disease in Changing Climates: Strategies of an Amphibian Pathogen.” Proceedings of the National Academy of Sciences of the United States of America 111: 7001–7006. 10.1890/06-1842.1 18589527

[ece373944-bib-0076] Woodhams, D. C. , J. McCartney , J. B. Walke , and R. Whetstone . 2023. “The Adaptive Microbiome Hypothesis and Immune Interactions in Amphibian Mucus.” Developmental & Comparative Immunology 145: 104690. 37001710 10.1016/j.dci.2023.104690 PMC10249470

[ece373944-bib-0077] Yang, D.‐S. , and B. H. Koo . 2016. “A Study on the Improvement Plan for a Habitat of ‘Gold‐Spotted Pond Frog ( *Pelophylax chosenicus* )’ in Danger of Regional Extinction in the Urban Area‐Case on the Abandoned Railroad Site on Su‐In Line.” Journal of the Korea Society of Environmental Restoration Technology 19, no. 2: 95–107.

